# Muscle satellite cell dysfunction in critical illness: niche biology, regenerative failure, and implications for recovery

**DOI:** 10.3389/fcell.2026.1984134

**Published:** 2026-09-29

**Authors:** Takafumi Ono, Atsushi Asakura

**Affiliations:** 1 Stem Cell Institute, University of Minnesota Medical School, Minneapolis, MN, United States; 2 Greg Marzolf Jr. Muscular Dystrophy Center and Department of Neurology, University of Minnesota Medical School, Minneapolis, MN, United States

**Keywords:** critical illness, intensive care unit–acquired weakness (ICU-AW), muscle satellite cells, myogenesis, Notch pathway, post-intensive care syndrome, skeletal muscle regeneration, stem cell niche

## Abstract

Adult skeletal muscle can regenerate. After muscle injury, muscle stem cells (satellite cells) can generate new muscle fibers. Under normal conditions, satellite cells are mitotically quiescent and located beneath the basal lamina of muscle fibers. In response to regenerative stimuli, satellite cells activate and proliferate as myogenic precursor cells. After several rounds of cell division, these precursor cells differentiate and fuse to form new muscle fibers. A subset of proliferating myogenic precursor cells returns to a quiescent state to replenish the satellite cell pool through self-renewal. Intensive care unit–acquired weakness (ICU-AW) affects many critically ill patients partly due to acute muscle loss. It is a major physical component of post-intensive care syndrome. Current explanations of ICU-AW focus on rapid protein breakdown and impaired energy metabolism during the early catabolic phase, but these mechanisms do not fully explain why some survivors fail to rebuild muscle once the initial catabolic stress has subsided. In this review, we argue that persistent weakness may also reflect defective muscle regeneration, with satellite cell dysfunction and niche abnormalities as plausible contributors. Recent serial-biopsy studies suggest that dysfunction can occur without stem-cell depletion: satellite cell number can be preserved even when differentiation or later proliferative capacity is impaired in culture. We first review the normal satellite cell niche, including the parent fiber, microvasculature and perivascular cells, extracellular matrix, resident immune and interstitial cells, and systemic signals, with separate consideration of the diaphragm. We then examine how critical illness affects these compartments and how cell-intrinsic defects may additionally impair regeneration. We use this evidence to develop three working hypotheses (niche-detached activation, abortive myogenesis, and niche locking) and treat them as testable models rather than recognized mechanisms. Finally, we consider how future studies might measure, target, and test these processes.

## 1 Introduction

Skeletal muscle is the most abundant tissue in the human body, accounting for approximately 30%–40% of total body weight. It is composed of multinucleated muscle fibers that contract to generate force and enable movement. In addition to its essential mechanical functions, skeletal muscle has remarkable regenerative capacity and can repair rapidly after severe damage from exercise, injury, or disease. Muscle satellite cells are a stem cell population essential for postnatal skeletal muscle growth and regeneration. Satellite cell nuclei account for approximately 2.5%–6% of the nuclei beneath the basal lamina in adult mouse muscle (Motohashi and Asakura, 2014). Satellite cells reside beneath the basal lamina and adjacent to the plasma membrane of muscle fibers, where they normally remain mitotically quiescent. Following muscle injury, satellite cells become activated and enter the cell cycle to proliferate and generate myogenic precursor cells; they then withdraw from the cell cycle, differentiate into myocytes, and fuse either with one another to form new myotubes or with existing myofibers to repair damaged muscle. During this regenerative process, a subset of satellite cells undergoes self-renewal to return to a quiescent state and replenish the satellite cell pool under normal physiological conditions (Wang and Rudnicki, 2012; Motohashi and Asakura, 2014). This self-renewal capacity, which maintains a population of satellite cells capable of responding to repeated episodes of muscle injury, represents a defining characteristic of these cells.

### 1.1 ICU-acquired weakness and recovery after critical illness

Many survivors of critical illness experience persistent muscle weakness after ICU discharge. ICU-acquired weakness (ICU-AW) is characterized by diffuse, symmetrical weakness that develops during critical illness and includes critical illness polyneuropathy, critical illness myopathy, and their overlap (Hermans et al., 2008; Stevens et al., 2009; Vanhorebeek et al., 2020). Commonly associated with prolonged mechanical ventilation, sepsis, multi-organ failure, and severe respiratory distress, ICU-AW is linked to prolonged hospitalization, delayed ventilator weaning, and increased mortality (Fan et al., 2014; Hermans et al., 2014). Weakness and impaired exercise capacity may persist for years (Herridge et al., 2011; Dinglas et al., 2017), contributing substantially to post-intensive care syndrome and reducing quality of life and independence in daily activities (Needham et al., 2012; Wieske et al., 2015; Inoue et al., 2024). Persistent inflammation, immunosuppression, and catabolism may further impair muscle recovery (Gentile et al., 2012; Mira et al., 2017a; Mira et al., 2017b). Recent work has examined these concepts in detail, including systematic reviews of risk factors, biomarkers, and possible treatments (Chadda and Puthucheary, 2024a; Chadda et al., 2024b). The COVID-19 pandemic has also highlighted the long-term burden of acquired weakness among survivors of prolonged critical illness (Taketa et al., 2024; Chuang et al., 2025; Egger et al., 2026). Most available data come from biopsy series, electrophysiology, and muscle ultrasound (Lad et al., 2020; Vanhorebeek et al., 2020; Elkalawy et al., 2023; Chen and Huang, 2024; Fuentes-Aspe et al., 2024; Umbrello et al., 2024). Despite its clinical significance, the mechanisms underlying ICU-AW remain incompletely understood because direct investigation of human skeletal muscle is limited. Diagnosis remains challenging, particularly in sedated or delirious patients, and distinguishing neuropathic from myopathic contributions is often difficult (Egger et al., 2024; Rodriguez et al., 2024). In addition to limb muscles, respiratory muscle weakness, particularly diaphragm dysfunction, can contribute to ventilator weaning failure, impaired cough, and increased susceptibility to aspiration pneumonia (Saccheri et al., 2020).

### 1.2 Why acute catabolism is not the whole story

Acute muscle loss during critical illness is well characterized and results primarily from increased protein degradation through the ubiquitin–proteasome and autophagy pathways, reduced protein synthesis, and inflammatory and hormonal signaling (Puthucheary et al., 2013). Myofibrillar proteins, particularly myosin, are disproportionately degraded, contributing to severe muscle weakness (Wollersheim et al., 2014). These acute catabolic mechanisms are well established and have been extensively reviewed (Zhou et al., 2014; Friedrich et al., 2015; Hermans and Van den Berghe, 2015; Schefold et al., 2020; Latronico et al., 2023). However, acute catabolism alone cannot fully explain the persistent weakness observed in many survivors (Intiso et al., 2022; Mart et al., 2026). Months to years after critical illness, muscle mass and function often recover incompletely, with fibrosis, collagen deposition, and fatty infiltration suggesting impaired restoration of normal muscle architecture (Dos Santos et al., 2016; Mart et al., 2026; Lees et al., 2025).

### 1.3 Early evidence for impaired regeneration after critical illness

Emerging evidence points to defective muscle regeneration as an important contributor to long-term recovery failure. Studies in humans and mice indicate that critical illness and sepsis can impair muscle satellite cell function, including proliferation, differentiation, metabolism, and mitochondrial activity (Dos Santos et al., 2016; Rocheteau et al., 2015; Schmitt et al., 2023). Although satellite cell numbers may remain relatively preserved, their regenerative capacity can remain compromised long after the acute catabolic phase has resolved. Recent human studies further suggest that impaired satellite cell function is associated with muscle weakness, atrophy, and aging (Krajčová et al., 2026). Thus, persistent muscle weakness after critical illness may reflect not only continued catabolism but also a failure of effective satellite cell–mediated muscle repair and regeneration (Mathur et al., 2025).

### 1.4 Aim of this review

Previous reviews have addressed several aspects. Some have followed sepsis-associated muscle wasting through proteolytic and inflammatory pathways, from experimental models to the bedside (Yoshihara et al., 2023); others have made the case for mitochondrial dysfunction as a shared mechanism in ICU-AW and critical illness myopathy (Klawitter et al., 2023); and one has placed the microcirculation and cardiovascular physiology at the center of the account (Mendelson et al., 2023). A recent, wide-ranging review summarized the biology and clinical obstacles of weakness after critical illness, including emerging single-cell data (Pierre et al., 2025a). None of these has been structured around the satellite cell and its niche, treating regeneration as a coordinated process carried out by multiple cell types within a defined tissue environment and asking how critical illness alters the conditions required for effective repair. The present review is structured around the satellite cell and its niche. It starts with the normal biology of the satellite cell and the components of its niche: the parent fiber, the small vessels and their associated mural cells, the extracellular matrix and basement membrane, the neighboring immune and fibro-adipogenic progenitors (FAPs), and the hormonal and metabolic signals reaching muscle from elsewhere in the body. The diaphragm is considered separately, since respiratory muscle differs from limb muscle. We then examine each compartment in turn for the effects of critical illness, alongside intrinsic defects in the satellite cell that may modulate those effects. We bring together human and animal evidence on how an acute injury leads to persistent impairment, and at the end of the relevant sections we propose three working hypotheses: niche-detached activation, abortive myogenesis, and niche locking. Each is stated as a testable prediction, not an established mechanism. This framework addresses the myopathic and regenerative component of persistent weakness; it does not account for the full neuropathic contribution to ICU-AW. The final sections consider how these processes might be assessed, potential interventions, and unresolved questions. Throughout the manuscript, findings demonstrated directly in critically ill patients are discussed separately from mechanisms inferred from animal, injury, aging, or other disease models.

## 2 The satellite cell and its niche in healthy muscle

### 2.1 The satellite cell and the course of regeneration

Adult skeletal muscle is repaired by a dedicated stem cell population termed satellite cells, which resides between the plasma membrane of the muscle fibers and the surrounding basal lamina (Figure 1). This sublaminar space forms the immediate niche of satellite cells. Satellite cells arise during development from Pax3- and Pax7-expressing myogenic progenitors of the dermomyotome and are involved in postnatal muscle growth and regeneration (Asakura and Tapscott, 1998; Asakura and Rudnicki, 2002). In resting adult muscle, satellite cells are retained in this anatomical position, remain quiescent, and express the transcription factor Pax7 (Relaix and Zammit, 2012; Yin et al., 2013). After muscle fiber injury, satellite cells become activated, express MyoD, and enter the cell cycle to proliferate, followed by induction of Myogenin during differentiation and fusion with one another to generate new multinucleated fibers or with existing fibers to repair skeletal muscle (Wang and Rudnicki, 2012). A subset of daughter cells retains Pax7 expression and returns to quiescence, replenishing the stem-cell pool through self-renewal for future repair (Dumont et al., 2015a). This balance between myogenic differentiation and self-renewal permits tissue repair without depleting the stem-cell reservoir (Figure 2; Bentzinger et al., 2012). Under specific experimental conditions in culture, satellite cells can also adopt osteogenic and adipogenic fates, highlighting their broader multipotent differentiation potential (Asakura et al., 2001; Komaki et al., 2004). In mouse skeletal muscle, side population cells distinct from satellite cells acquired myogenic potential after coculture with myoblasts (Asakura et al., 2002b). Genetic ablation studies of Pax7-positive cells show that satellite cells are required for effective regeneration; without them, injured muscle shows defective repair with increased fibrotic healing (Relaix and Zammit, 2012). Satellite cells do not act in isolation; their function depends on molecular signals and physical cues from the surrounding tissue environment, making the niche an integral component of regeneration (Figure 1) (Yin et al., 2013).

**FIGURE 1:**
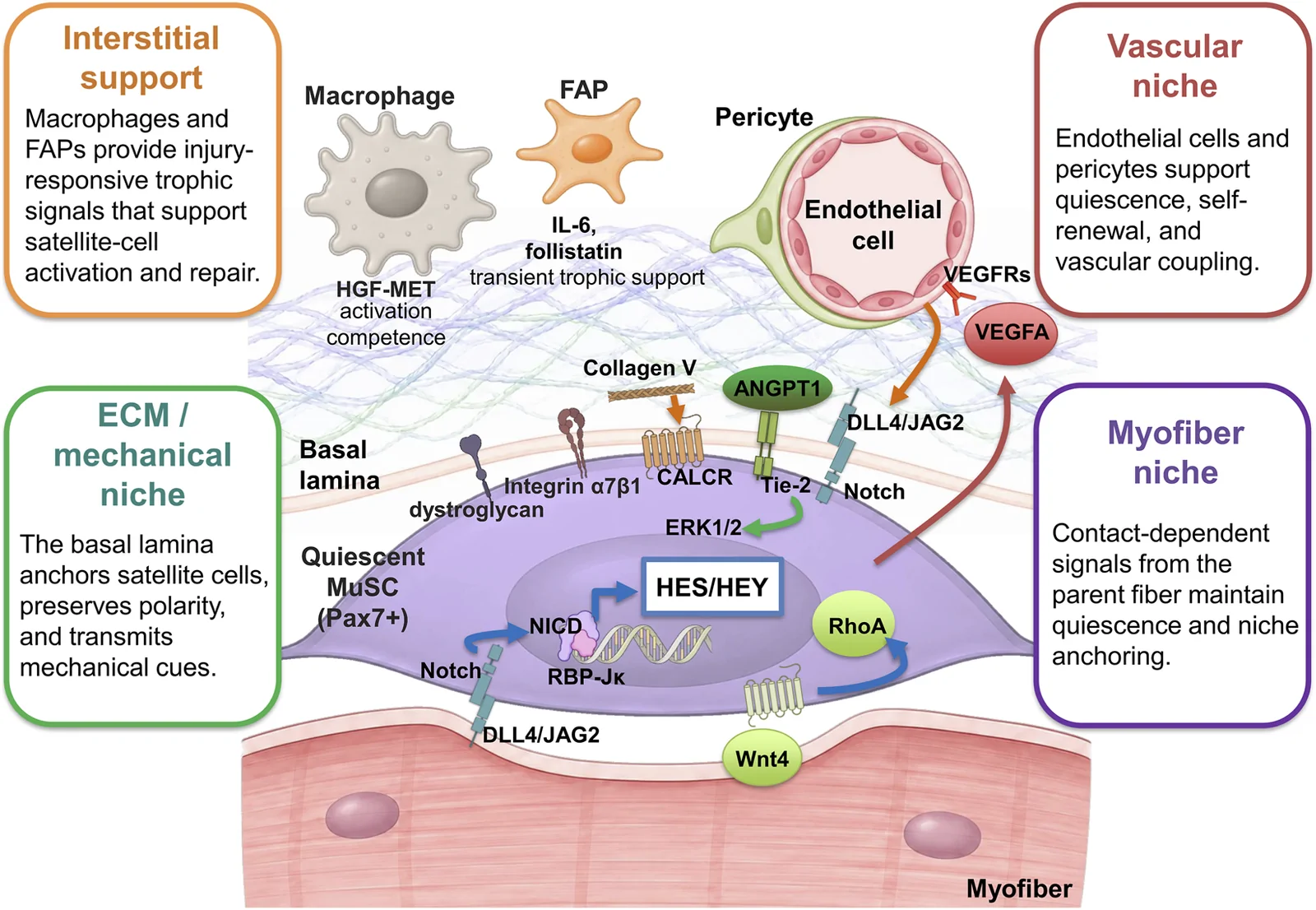
The healthy muscle satellite cell niche. In resting skeletal muscle, a quiescent muscle satellite cell (MuSC) resides between the myofiber plasma membrane and the basal lamina, where it receives spatially organized signals from the parent fiber, extracellular matrix (ECM), microvasculature, and interstitial support cells. Myofiber-derived DLL4 provides an important Notch input in the resting niche, while endothelial DLL4 can provide an additional juxtavascular Notch signal. Notch activation generates NICD–RBP-Jκ–HES/HEY signaling and maintains quiescence. Myofiber-derived Wnt4 acts through RhoA to support the satellite cell’s stiffness and niche retention. The basal lamina anchors the cell through integrin α7β1 and dystroglycan, while satellite cell-derived collagen V engages the calcitonin receptor (CALCR) to reinforce quiescence. MuSC-derived VEGFA promotes endothelial recruitment and helps maintain vascular proximity. Angiopoietin-1 (ANGPT1) acts through Tie-2 and ERK1/2 in an autocrine or paracrine manner to promote return to quiescence and self-renewal. Macrophage-derived hepatocyte growth factor (HGF) and fibro-adipogenic progenitor (FAP)-derived interleukin-6 (IL-6) and follistatin are injury-responsive trophic signals that support satellite cell activation and repair. Signals from the niche regulate satellite cell quiescence and activation according to tissue state.

**FIGURE 2:**
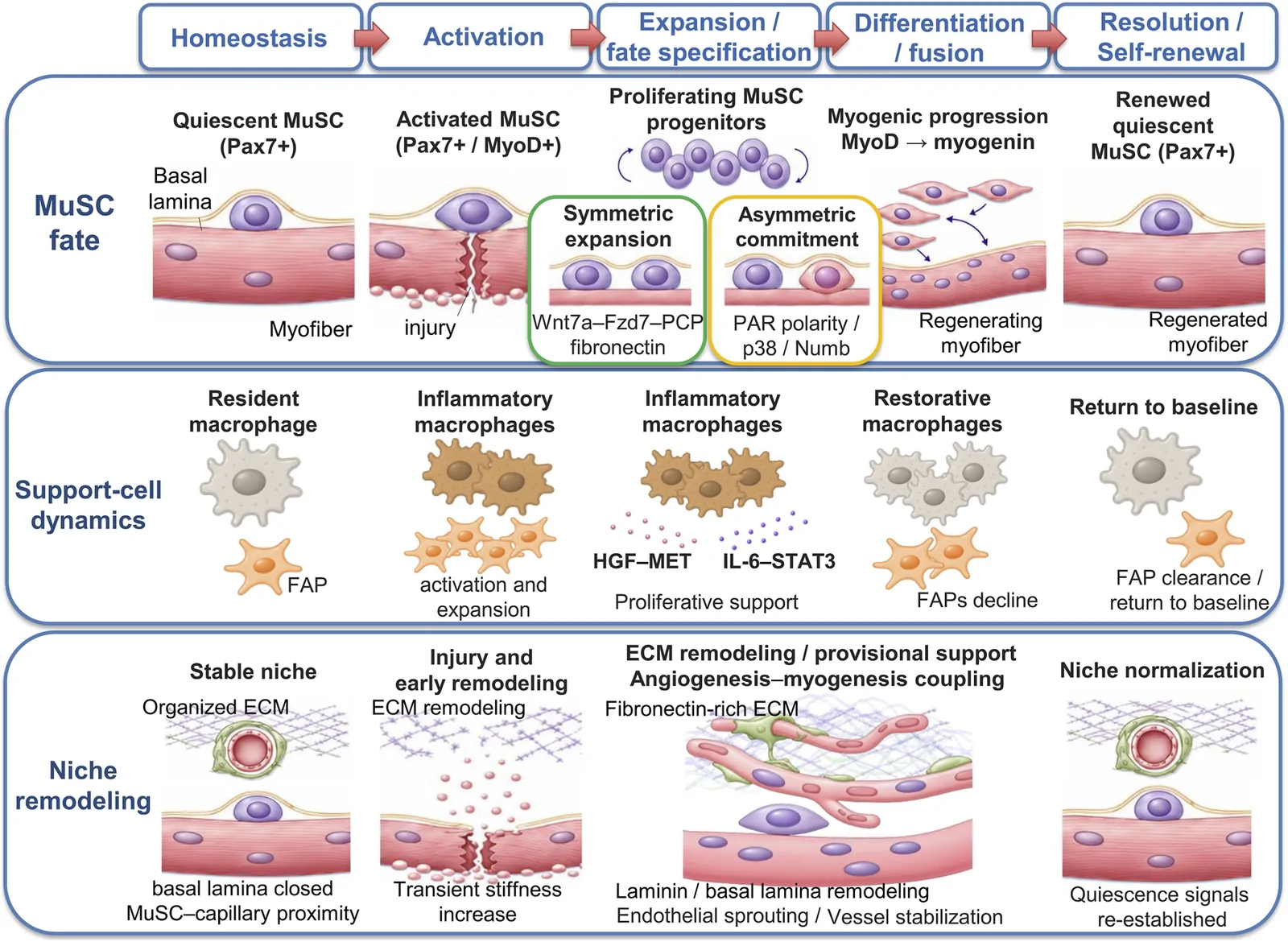
Coordinated satellite cell and niche dynamics during skeletal muscle regeneration. Following myofiber injury, quiescent Pax7-positive satellite cells activate, induce MyoD, and enter a proliferative phase in which cell fate is specified between stem-cell expansion and myogenic commitment. The inset summarizes this fate specification. Wnt7a–Fzd7 signaling through the planar cell polarity (PCP) pathway, supported by fibronectin, promotes symmetric expansion, whereas polarity cues involving the PAR complex, p38, and Numb contribute to asymmetric fate specification. In parallel, inflammatory macrophages and fibro-adipogenic progenitors (FAPs) expand and provide transient signals that support satellite cell activation and proliferation. As myogenic cells progress toward differentiation and fusion, macrophages shift toward a restorative state, FAPs decline, and angiogenesis becomes coupled to new muscle formation. Extracellular matrix (ECM) and basal lamina remodeling provide a provisional regenerative niche before matrix organization, vascular proximity, and quiescence signals are restored. A subset of satellite cells returns to quiescence and replenishes the stem-cell pool. These phases overlap *in vivo*; the columns indicate dominant processes rather than discrete temporal boundaries.

### 2.2 The parent fiber and Notch signaling

The fiber to which a satellite cell is attached is the first and most immediate component of its niche (Figure 1). Canonical Notch signaling actively maintains quiescence, and inhibiting this pathway causes satellite cells to exit quiescence and undergo premature differentiation, progressively depleting the stem-cell pool (Bentzinger et al., 2012; Bjornson et al., 2012; Mourikis et al., 2012). The primary Notch ligand on the parent fiber membrane is Delta-like 4 (DLL4), which engages Notch receptors (mainly NOTCH1 and NOTCH3) on the apposed satellite cell surface (Yin et al., 2013; Eliazer et al., 2022). Delta-like 1 (DLL1) is mainly expressed by activated and differentiating myogenic cells during regeneration, where it signals through NOTCH2 to sustain self-renewal in neighboring satellite cells rather than maintain quiescence in the resting niche (Yartseva et al., 2020). Ligand-receptor engagement triggers γ-secretase-mediated cleavage of the Notch intracellular domain (NICD), which translocates to the nucleus and forms a transcriptional complex with the DNA-binding factor RBP-Jκ and the co-activator MAML. This complex induces the bHLH repressors HES1, HEY1, and HEYL, which in turn repress MyoD and other pro-myogenic genes, thereby maintaining the satellite cell in a non-committed state. Conditional deletion of RBP-Jκ in adult satellite cells causes spontaneous activation, premature differentiation, and progressive pool depletion, confirming the requirement for canonical Notch signaling to maintain quiescence (Bjornson et al., 2012; Mourikis et al., 2012).

In uninjured mouse muscle, the myofiber appears to be the major source of DLL4 captured by quiescent satellite cells (Eliazer et al., 2022). This does not exclude other cellular sources in different tissue states; Section 2.3 discusses endothelial DLL4 separately as part of the vascular niche. Throughout the length of the multinucleated fiber, DLL4 is distributed heterogeneously, and local ligand abundance is associated with differences in Pax7 expression levels and quiescent-state depth across the satellite cell population (Eliazer et al., 2022). The satellite cell itself also contributes to this regulatory architecture: Notch signaling drives local deposition of collagen V, which engages the calcitonin receptor (CALCR) on the same cell and reinforces quiescence (Baghdadi et al., 2018). The fiber further supplies Wnt4, which increases RhoA activity and helps maintain the cell stiffness and niche retention of quiescent satellite cells (Eliazer et al., 2019). These signals may be altered in critical illness, because injury to the fiber or changes in its surface and secretory profile can remove signals that normally help maintain the satellite cell in a stable, regeneration-competent state.

### 2.3 The vascular and perivascular niche

Satellite cells are located close to capillaries. In human muscle, most are within a few microns of the nearest microvessel, and this distance increases with age in parallel with declining regenerative capacity (Christov et al., 2007; Nederveen et al., 2016; Verma et al., 2018). The proximity is functional as well as anatomical, and it supports bidirectional signaling between satellite cells and the vascular wall. Endothelial cells can provide DLL4- and JAG2-dependent Notch signals to nearby satellite cells, while satellite cell-derived VEGFA supports endothelial chemotaxis, survival, and capillary growth (Verma et al., 2018; Tanaka et al., 2026). Satellite cells also express the VEGF receptor FLT1, and VEGFA-FLT1-AKT1 signaling promotes their survival during regeneration, adding a cell-autonomous component to this vascular cross-talk (Verma et al., 2018; Verma et al., 2024). Angiopoietin-1 (ANGPT1), released by cells in the satellite cell neighborhood, acts through Tie-2 on myogenic precursors and promotes return to quiescence and self-renewal through ERK1/2 signaling (Abou-Khalil et al., 2009). A distinct AKT–FOXO pathway regulates FOXO localization, while FOXO3 promotes quiescence during self-renewal in part by increasing Notch1 and Notch3 expression (Brunet et al., 1999; Gopinath et al., 2014). These pathways should therefore not be collapsed into a single Tie-2–AKT–FOXO3 cascade. Angiogenesis occurs alongside the expansion of myogenic progenitors during muscle regeneration, and restorative macrophages help coordinate these processes. Macrophages were the main source of oncostatin M (OSM) transcripts among the cell populations examined in regenerating mouse muscle. In human three-dimensional cocultures, OSM blockade reduced the effects of restorative macrophages on capillary elongation and markedly inhibited lumen formation and myotube formation (Latroche et al., 2017).

Pericytes, perivascular mural cells embedded within the capillary basement membrane, wrap around capillaries, contribute to vessel stability, and form a second cellular layer of the vascular niche (Murray et al., 2017). Pericytes are functionally heterogeneous: type-2 pericytes (NG2-positive, Nestin-positive) display myogenic potential and contribute to regeneration, whereas type-1 pericytes (NG2-positive, Nestin-negative) can differentiate into adipogenic and fibrogenic cells and may contribute to fatty infiltration when repair fails (Birbrair et al., 2013). Pericytes can also serve as an alternative source of myogenic progenitors, fusing into existing fibers and contributing to the satellite cell compartment in postnatal muscle (Dellavalle et al., 2011; Birbrair et al., 2013). Within this myovascular niche, pericytes drive postnatal fiber growth and supply ANGPT1 and insulin-like growth factor 1 (IGF-1) to maintain satellite cell quiescence and support myogenic differentiation, respectively (Kostallari et al., 2015). The microvascular bed is therefore not merely a conduit for oxygen and nutrients but an active component of the niche, organized as an endothelial–pericyte–basal lamina–satellite cell unit. Its failure in critical illness has consequences that extend well beyond impaired perfusion.

### 2.4 The extracellular matrix and mechanical cues

Between the satellite cell and the surrounding tissue lie the basal lamina and the broader extracellular matrix, which together serve as both a structural scaffold and a signaling platform (Loreti and Sacco, 2022). The basal lamina does more than enclose the cell. It is composed principally of laminin-211, collagen IV, perlecan, and nidogen, and these components engage receptors on the satellite cell surface: laminin binds integrin α7β1 and the dystroglycan complex, attachments that anchor the cell within its niche and transmit polarity and survival signals. The matrix is actively remodeled during repair, with new laminin deposited at the niche as the cell divides, and this remodeling is required for satellite cell self-renewal (Rayagiri et al., 2018). Individual matrix components exert distinct regulatory effects: collagen VI contributes to the mechanical properties of the niche and is required for normal self-renewal and regeneration (Urciuolo et al., 2013), while fibronectin serves as the substrate for presentation of the pool-expanding Wnt7a signal, and its progressive loss from aged muscle impairs regenerative capacity (Lukjanenko et al., 2016). Notch signaling also promotes the localized deposition of collagen V, which binds to CALCR on the same cell, thereby enhancing the quiescent state (Baghdadi et al., 2018).

Satellite cells sense matrix stiffness through the same integrin and dystroglycan attachments. After injury, the matrix stiffens, and this mechanical change is transmitted through integrins and the actin cytoskeleton to the Hippo-pathway effectors YAP and TAZ, which translocate to the nucleus and maintain the satellite cell in an activated state; when matrix stiffness fails to resolve, satellite cells remain activated rather than returning to quiescence (Silver et al., 2021). This observation is particularly relevant to the chronic phase of critical illness, in which a persistently fibrotic matrix may keep satellite cells in an inappropriate activation state long after the initiating injury has resolved.

### 2.5 Immune and interstitial cells

Regeneration depends on the coordinated activity of multiple supporting cell populations in addition to the satellite cell itself (Johnson et al., 2023). In mouse muscle injury models, inflammatory Ly6C-positive monocytes are recruited to the damaged muscle, clear necrotic debris by phagocytosis, and differentiate into Ly6C-negative macrophages with a restorative, anti-inflammatory phenotype. Satellite cell proliferation peaks while pro-inflammatory macrophages dominate the injury site, and myogenic differentiation begins as the macrophage population transitions toward the restorative state; the timing of this phenotypic switch therefore sets the pace of repair (Arnold et al., 2007; Tidball, 2017; Dort et al., 2019).

Macrophages have functions beyond debris clearance. A specific macrophage subset promotes satellite cell proliferation through hepatocyte growth factor and its receptor Met (Koike et al., 2025). Cytokines in the regenerative milieu also act directly on satellite cells, and interleukin-6 (IL-6) signals through STAT3 as an essential regulator of satellite cell-mediated growth (Serrano et al., 2008). Macrophages can additionally function as a transient niche-like support population by secreting nicotinamide phosphoribosyltransferase, which maintains satellite cell cycling during regeneration (Ratnayake et al., 2021).

FAPs are mesenchymal cells in the muscle interstitium that provide transient support during regeneration. In mice, they are commonly identified by PDGFRα and Sca-1 expression. FAPs expand after injury and release trophic factors, including IL-6 and follistatin (Wosczyna and Rando, 2018). During successful repair, the expanded FAP population declines during the resolution phase, in part through tumor necrosis factor-α-mediated apoptosis. When this clearance fails, FAPs persist and can contribute to fibrotic and adipogenic remodeling (Lemos et al., 2015).

Regulatory T cells (Tregs) accumulate in injured muscle during the later phase of regeneration and help bring the inflammatory response toward resolution. Their most well-established direct trophic signal to the myogenic compartment is amphiregulin, an EGFR ligand that acts on satellite cells and improves muscle repair in experimental models (Burzyn et al., 2013). Local Treg accumulation depends on interleukin-33 (IL-33) supplied in part by FAPs; in aged muscle, impaired IL-33-dependent recruitment is accompanied by poorer repair (Kuswanto et al., 2016). The myokine meteorin-like, induced after injury, supports restorative macrophage polarization and increases IGF-1 production through a STAT3-dependent pathway, thereby supporting satellite cell proliferation (Baht et al., 2020).

Satellite cell activity, then, depends on the identity, timing, and resolution of the surrounding support-cell response as well as on the intrinsic stem-cell program (Figure 2). When inflammation fails to resolve appropriately, as may occur in chronic critical illness, this temporal coordination and the timely clearance of FAPs in particular may be disrupted.

### 2.6 How the satellite cell decides its fate

When a satellite cell divides, it can self-renew, generate committed progeny, or undergo both processes simultaneously; the balance among these outcomes determines whether the stem-cell pool is maintained (Figure 2, inset) (Almada and Wagers, 2016). Some divisions are asymmetric, producing two daughters with different fates: one retains stem-cell identity, while the other commits to differentiation. Satellite cells with no recorded history of Myf5 expression in Myf5-Cre/ROSA26-YFP lineage-tracing mice occupy the apex of this hierarchy. When such a cell divides along the apical–basal axis of the fiber, the daughter that retains contact with the basal lamina remains YFP-negative and preserves stem-cell identity, while the apical daughter, displaced toward the fiber, becomes YFP-positive and commits to differentiation (Kuang et al., 2007).

This asymmetry is established by division orientation and by signaling molecules asymmetrically distributed between the two daughters. The PAR complex (PARD3, PARD6, and aPKC) anchors to one side of the cell cortex through interaction with the dystrophin–glycoprotein complex and the kinase MARK2, generating cortical polarity along the apical–basal axis. p38α/β mitogen-activated protein kinase activity is enriched in the committed daughter, where it phosphorylates BAF60c, a subunit of the SWI/SNF chromatin-remodeling complex; phosphorylated BAF60c is recruited to MyoD target loci, opens their chromatin, and activates the myogenic transcriptional program (Simone et al., 2004; Forcales et al., 2012; Troy et al., 2012). The cell-autonomous Notch inhibitor Numb has also been implicated in asymmetric satellite cell division (Kuang et al., 2007; Almada and Wagers, 2016).

Symmetric divisions, which expand the stem-cell pool rather than deplete it, operate through a different signaling axis. Wnt7a, secreted by neighboring myofibers, engages the Frizzled-7 (Fzd7) receptor on the satellite cell and activates the non-canonical planar cell polarity pathway, reorienting the mitotic spindle parallel to the fiber and producing two daughters with equivalent stem-cell potential (Le Grand et al., 2009; Bentzinger et al., 2013). Fibronectin in the basement membrane acts as a substrate for Wnt7a presentation and is required for efficient planar-cell-polarity-mediated symmetric division (Bentzinger et al., 2013). Polarity itself depends on proteins not traditionally associated with stemness: dystrophin is expressed in satellite cells and anchors the polarity machinery, and its loss misorients the divisions and biases them away from renewal, providing a mechanistic basis for the functional impairment of the satellite cell pool in muscular dystrophy (Dumont et al., 2015b). How faithfully the satellite cell interprets these polarity, mechanical, and molecular cues, and how reliably the niche supplies them, determines whether an injury is followed by adequate stem-cell renewal or by progressive depletion of regenerative capacity (Feige et al., 2018).

### 2.7 Metabolism and the epigenetic state

A quiescent satellite cell maintains a low metabolic state that relies predominantly on fatty-acid oxidation, and this metabolic configuration is tightly coupled to its epigenetic state. As the cell exits quiescence and activates, it shifts toward glycolysis; the decline in fatty-acid oxidation lowers intracellular NAD + levels, and because the deacetylase SIRT1 is NAD + -dependent, its activity drops. The resulting hyperacetylation of histone H4 at lysine 16 (H4K16ac) across muscle-specific gene loci permits transcriptional activation of the myogenic program, so that exit from quiescence is simultaneously a metabolic and a transcriptional event (Ryall et al., 2015). Fatty-acid oxidation in satellite cells therefore exerts a regulatory rather than purely bioenergetic role, shaping stem-cell function by modulating metabolic flux and the acetylation of regulatory proteins (Yue et al., 2025). Activation also requires a transient burst of autophagy, which meets the bioenergetic demand of the quiescence-to-activation transition by recycling intracellular components; pharmacological inhibition of autophagy at this stage stalls satellite cell activation (Tang and Rando, 2014). Mitochondria are central to these transitions, since their content, morphology, and dynamic behavior influence whether a satellite cell remains quiescent, commits to differentiation, or returns to self-renewal (Bhattacharya and Scimè, 2020). These linkages between metabolism, mitochondrial function, chromatin state, and fate are particularly relevant to critical illness, which perturbs all of these systems simultaneously; under such conditions, machinery that normally fine-tunes satellite cell fate may instead promote maladaptive activation, impaired self-renewal, or failed differentiation.

### 2.8 A special case: the diaphragm

Most of the biology described in the preceding sections applies to limb muscle. In the ICU, however, the diaphragm is a muscle of major clinical importance during ventilator weaning, and it differs biologically from limb muscle. Mechanical ventilation unloads the diaphragm, and within one to 2 days of controlled ventilation, human diaphragm fibers show rapid disuse atrophy (Levine et al., 2008). Diaphragm weakness develops rapidly and progresses during ventilation (Jaber et al., 2011), with proteasome activation correlating with the loss of fiber force in critically ill patients (Hooijman et al., 2015). Several molecular mechanisms have been characterized. Mechanical ventilation increases mitochondrial reactive oxygen species in the diaphragm, and oxidative stress activates proteolytic pathways, with crosstalk between autophagy and oxidative signaling setting the rate of myofibrillar breakdown (Smuder et al., 2018). Calcium-activated calpain proteases are required for both the weakness and the associated mitochondrial dysfunction (Hyatt et al., 2021), and a shift in mitochondrial dynamics toward fission contributes independently to diaphragm weakness (Dridi et al., 2023). Findings differ among studies: diaphragm atrophy and weakness can also develop in critically ill patients without measurable mitochondrial dysfunction, indicating that multiple pathways can produce the same functional endpoint (van den Berg et al., 2017). Ventilator-induced diaphragm dysfunction has therefore been extensively reviewed (Powers et al., 2013; Peñuelas et al., 2019), and ICU-acquired diaphragm weakness is common and prognostically significant (Dres et al., 2017).

Diaphragm satellite cells differ from many limb-muscle satellite cells in the regulation of Pax3 expression. Pax3 is expressed in diaphragm myogenic cells but is restricted to subsets of satellite cells in several other muscles (Stuelsatz et al., 2012). In mouse diaphragm satellite cells, alternative polyadenylation generates Pax3 transcripts with short 3′untranslated regions that lack miR-206 target sites. The longer, miR-206-sensitive transcripts predominate in the limb satellite cells examined (Boutet et al., 2012). This establishes a difference in post-transcriptional regulation, although it does not demonstrate dependence on separate upstream activation signals.

Aging also affects diaphragm satellite cells. In male mice, satellite cell abundance was lower at 22 months than at 6 months. However, satellite cell depletion did not alter diaphragm fiber size, myonuclear number, or extracellular matrix content during 4 weeks of hypoxia at either age (Thomas et al., 2022). These results do not establish that diaphragm satellite cells are more susceptible to age-related depletion than limb satellite cells, or that they are dispensable for repair after injury.

Responses during mechanical ventilation require separate consideration. Satellite cells isolated from mouse diaphragms after 6 h of ventilation showed reduced proliferation and increased differentiation in culture (Ding et al., 2021). Fibrosis has been reported in diaphragms from ventilated critically ill patients (Shi et al., 2023). Single-nucleus profiling of rabbit diaphragms with and without 24 h of mechanical ventilation identified changes in stromal and immune cell transcriptional profiles (Li et al., 2024). Satellite cell transplantation improved diaphragm function in dystrophic mice (Azzag et al., 2024), but its effects after critical illness remain to be established.

## 3 How critical illness disrupts the niche

Critical illness can disturb multiple signals the satellite cell draws from its niche. The niche can be examined compartment by compartment, but the compartments do not fail one at a time. These disturbances may occur together, and their combined effects may impair recovery.

### 3.1 Microvascular failure and the satellite cell’s loss of vascular contact

Critical illness, especially sepsis, often involves endothelial dysfunction. The vessel wall becomes activated and leaky: the glycocalyx lining the lumen is shed, leukocytes adhere, barrier integrity is lost, and fluid and protein escape into the interstitium (Goldenberg et al., 2011; Joffre et al., 2020). Adherens junctions that normally seal the endothelium, organized around VE-cadherin, disassemble as VE-cadherin is internalized and permeability rises (Dejana et al., 2008). The Angiopoietin–Tie-2 axis, which normally maintains vascular quiescence and stability, shifts toward an Angiopoietin-2 (ANGPT2)-dominant state during critical illness: ANGPT2, stored in the endothelium and released on activation, competes with ANGPT1 for the Tie-2 receptor, so Tie-2 signaling is reduced, the endothelium is destabilized, and the leak worsens (Sack et al., 2020; Chi et al., 2024). This results in interstitial edema, which increases the distance oxygen and signaling molecules must travel to reach cells embedded in the tissue (Swartz and Fleury, 2007; Wiig and Swartz, 2012). The pericytes that invest the capillaries are themselves lost or dysfunctional in sepsis; lipopolysaccharide drives pericyte loss and microvascular failure, in part by disturbing the same Angiopoietin–Tie-2 and Notch signals that normally stabilize the vessel, and preserving the pericytes protects the barrier (Zeng et al., 2016; Zhang et al., 2023). For the satellite cell, this has two consequences at once. As described in Section 2.3, satellite cells normally sit within a few microns of a capillary and receive quiescence and survival cues from endothelial cells and pericytes. When the microcirculation fails, the cell may lose access to those cues while interstitial edema increases the effective diffusion distance between the satellite cell and its vascular partners. The vascular component of the niche may therefore fail as both a signaling source and a structural anchor. This concurrent loss has not been shown directly in the muscle of critically ill patients, and we offer it as an inference from the vascular biology rather than as an established fact. Under this hypothesis, microcirculatory dysfunction may contribute to several of the disturbances considered below.

### 3.2 Inflammatory signals reaching the satellite cell

The inflammatory milieu of critical illness does not stop at the fiber; it reaches the satellite cell and changes its behavior. Tumor necrosis factor, signaling through p38α to a Polycomb complex, drives the methyltransferase Ezh2 to lay repressive marks across the Pax7 locus and silence it, tying inflammation directly to the epigenetic control of the regenerative program (Palacios et al., 2010). Bacterial lipopolysaccharide blocks myogenic differentiation through Toll-like receptor 4 and nuclear factor-κB, and it pushes the fiber into catabolism through the same receptor by engaging the proteasome and autophagy together (Doyle et al., 2011; Ono and Sakamoto, 2017). Several of these cytokines are beneficial during an acute response but harmful when signaling is sustained. Interferon-γ is needed for efficient regeneration, yet a persistent signal resets myogenic fate by recruiting JARID2 and the Polycomb complex to muscle gene promoters and shutting them down (Cheng et al., 2008; Londhe and Davie, 2013). IL-6 and STAT3 also have stage-dependent effects. STAT3 promotes myogenic progression, whereas conditional Stat3 deletion increases satellite cell expansion but impairs differentiation. Transient JAK–STAT inhibition can promote expansion and improve repair in experimental models (Muñoz-Cánoves et al., 2013; Price et al., 2014; Tierney et al., 2014). Interleukin-1 (IL-1) and tumor necrosis factor act on myoblasts partly through cyclooxygenase-2 and the prostaglandins it makes, adding to the catabolic drive (Li et al., 2009; Alvarez et al., 2020). The alarmin HMGB1, released from stressed and dying cells, lowers Pax7 and impedes regeneration, and blocking it prevents wasting in animal models (Li et al., 2021; Ho et al., 2023). Sustained nuclear factor-κB activity disrupts satellite cell homeostasis and, by dysregulating Pax7 in the muscle microenvironment, drives cachexia-related wasting (He et al., 2013; Straughn et al., 2019), and sepsis can also push muscle toward atrophy and apoptosis through PLK1-AKT signaling (Cao et al., 2021). As reviewed elsewhere, the same cytokines that organize a normal acute response can impair satellite cell-mediated repair when their signaling persists (Londhe and Guttridge, 2015). Persistent critical illness, with its chronic low-grade inflammatory state, may therefore provide precisely the setting in which otherwise adaptive cytokine signals become maladaptive.

### 3.3 Neuroendocrine and metabolic derangement

Critically ill patients experience high glucocorticoid activity, whether from the stress response or treatment, and glucocorticoids strongly regulate muscle mass (Braun and Marks, 2015). They act in part through the glucocorticoid receptor and FOXO1, which together induce the atrophy ligase MuRF1; MuRF1 directly ubiquitinates myosin heavy chain and targets it for proteasomal degradation, and suppressing both MuRF1 and atrogin-1 prevents glucocorticoid-driven atrophy in cultured muscle (Clarke et al., 2007; Waddell et al., 2008; Castillero et al., 2013b). This myosin degradation provides a mechanistic link to the selective thick-filament loss that characterizes critical illness myopathy (Wollersheim et al., 2014). A further level of regulation operates on the same pathway, since the nuclear receptor PPARβ/δ regulates the glucocorticoid- and sepsis-induced activation of FOXO1 (Castillero et al., 2013a). Glucocorticoids can also misdirect repair: in a spinal cord injury model, a transient glucocorticoid surge was sufficient to redirect muscle regeneration toward heterotopic ossification, an example, outside the ICU setting, of how an endocrine stress can reshape regenerative fate (Alexander et al., 2024). Hypothalamic-pituitary-adrenal axis function is itself frequently altered during critical illness, sometimes manifesting as critical illness-related corticosteroid insufficiency (Téblick et al., 2019; Sobolewska et al., 2024). A further set of signals comes from the TGF-β family. Myostatin restrains satellite cell activation and self-renewal and, acting on myoblasts, lowers MyoD to block differentiation; it also links the glucocorticoid signal to satellite cell dysfunction by suppressing Akirin1 (Langley et al., 2002; McCroskery et al., 2003; Dong et al., 2013). A mediator identified specifically in this setting is SPSB1, an E3-ligase adaptor induced in myocytes by tumor necrosis factor, IL-1β, and IL-6. SPSB1 ubiquitinates and destabilizes the type II TGF-β receptor, and because TβRII-Akt-Myogenin signaling is itself required for differentiation, this loss of receptor impairs myogenesis. SPSB1 is elevated in the muscle of both septic mice and patients with ICU-acquired weakness, and knocking it down in mice lessens sepsis-induced atrophy, supporting a role for SPSB1 in inflammation-associated muscle dysfunction (Li et al., 2023). Catabolic and TGF-β-related signals therefore act through a shared set of effectors, including the FOXO-regulated ubiquitin ligases MuRF1 and atrogin-1, which promote proteasomal degradation during muscle wasting (Sandri et al., 2004; Bodine and Baehr, 2014). FOXO factors also regulate the expression of autophagy-related genes (Milan et al., 2015).

Metabolic abnormalities may also impair repair. Insulin resistance and hyperglycemia are common in critical illness. Excess glucose slows satellite cell proliferation, and advanced glycation end-products drive atrophy and insulin resistance through FOXO1 (van den Berghe et al., 2001; Furuichi et al., 2021; Du et al., 2023). Studies of diabetes provide further evidence of impaired muscle regeneration, with mechanisms ranging from altered Notch signaling in type 1 to satellite cell impairment by raised extracellular adenosine in type 2, showing that a disordered metabolic environment can impair the regenerative compartment on its own (D'Souza et al., 2016; Han et al., 2022; Espino-Gonzalez et al., 2024). Nutrition interacts with these processes in ways that may depend on timing and dose. In a large trial, early parenteral nutrition did not protect muscle and, if anything, tolerating an early macronutrient deficit was associated with less acquired weakness (Casaer et al., 2011; Hermans et al., 2013), and a later trial of higher protein dosing in patients at high nutritional risk found no benefit, with a signal of harm in the sickest (Heyland et al., 2023). One plausible explanation is autophagy: feeding early suppresses the autophagic clearance that critically ill muscle relies on to remove damaged components, and this autophagy deficiency has been documented in the muscle of both patients and animals (Derde et al., 2012; Vanhorebeek et al., 2023). Because quiescent satellite cells rely on autophagy for proper activation (Section 2.7), suppressing autophagy in the acute phase is another way the regenerative compartment may be compromised.

### 3.4 Matrix remodeling and the shift in interstitial cells

Circulating matrix metalloproteinases change in an enzyme-specific manner during sepsis. Compared with non-septic ICU patients, patients with sepsis had higher plasma MMP-8 and lower MMP-9 concentrations (Martin et al., 2014). In a separate sepsis cohort, higher plasma MMP-3 and tissue inhibitor of metalloproteinases-1 concentrations were associated with shock and mortality (Jones et al., 2022). These systemic findings do not establish changes in MMP activity or extracellular matrix remodeling within skeletal muscle.

FAPs produce a transient extracellular matrix scaffold that supports satellite cell-mediated regeneration, in addition to the trophic support described in Section 2.5. Their persistence and differentiation depend on the inflammatory environment. In mouse muscle, macrophage-derived TNF promotes FAP apoptosis during acute repair. In chronically injured mdx muscle, macrophage-derived TGF-β1 suppresses this apoptosis and promotes FAP differentiation into matrix-producing cells (Lemos et al., 2015). In a separate mouse model, denervation caused FAP accumulation and persistent STAT3–IL-6 signaling that contributed to muscle fiber atrophy and fibrosis (Madaro et al., 2018). These mechanisms may contribute to muscle fibrosis during critical illness, but their persistence and functional consequences in survivors require direct investigation.

### 3.5 The neuromuscular junction and denervation

In patients with critical illness polyneuropathy, impaired motor axonal function can functionally denervate muscle, and denervation is closely linked to satellite cell biology. The neuromuscular junction is maintained by reciprocal signaling between nerve and fiber: nerve-derived agrin acts on the muscle co-receptor LRP4 and the kinase MuSK to cluster acetylcholine receptors on the postsynaptic membrane, and on the muscle side the junction is supported in part by satellite cells that supply fresh myonuclei to the synaptic region. Depleting satellite cells impairs neuromuscular junction regeneration, and their gradual loss drives the junctional breakdown seen with aging, indicating that satellite cells help maintain the synapse, not only the fiber (Liu et al., 2015; Liu et al., 2017). Satellite cells can retain regenerative potential for a considerable period after denervation, at least initially (Wong et al., 2021). Still, their response to a disturbed junction weakens with age (Larouche et al., 2021). Denervation alters the fiber’s secretome in ways that influence the satellite cell’s progression through its lineage (Henze et al., 2024). In patients with both polyneuropathy and critical-illness inflammation, these effects do not occur in isolation; loss of neural input further removes a signal on which satellite cells depend, while exacerbating the vascular, matrix, and metabolic disturbances.

These findings suggest a first working hypothesis. We put it forward as a hypothesis to be tested; we have not demonstrated the mechanism. Normally, the signals that keep a satellite cell quiescent and then guide it through repair come largely from an intact niche: Notch and Wnt4 from the fiber, ANGPT1, VEGF, and Notch ligands from the vessels and pericytes, mechanical and biochemical cues from the matrix, and the timed signals of resident immune and interstitial cells. Critical illness may weaken or desynchronize these signals while exposing the satellite cell to sustained inflammatory, endocrine, and metabolic cues that favor activation and catabolism. A satellite cell in this setting may therefore be driven to leave quiescence and activate in a niche that can no longer supply what orderly progression requires, an activation uncoupled from its supporting environment. We call this niche-detached activation (Figure 3). The idea is testable: it predicts that satellite cells in muscle from critically ill patients enter the cell cycle while the vascular, matrix, and immune components of their niche are still disrupted, and that the mismatch, rather than either defect alone, impairs repair.

**FIGURE 3:**
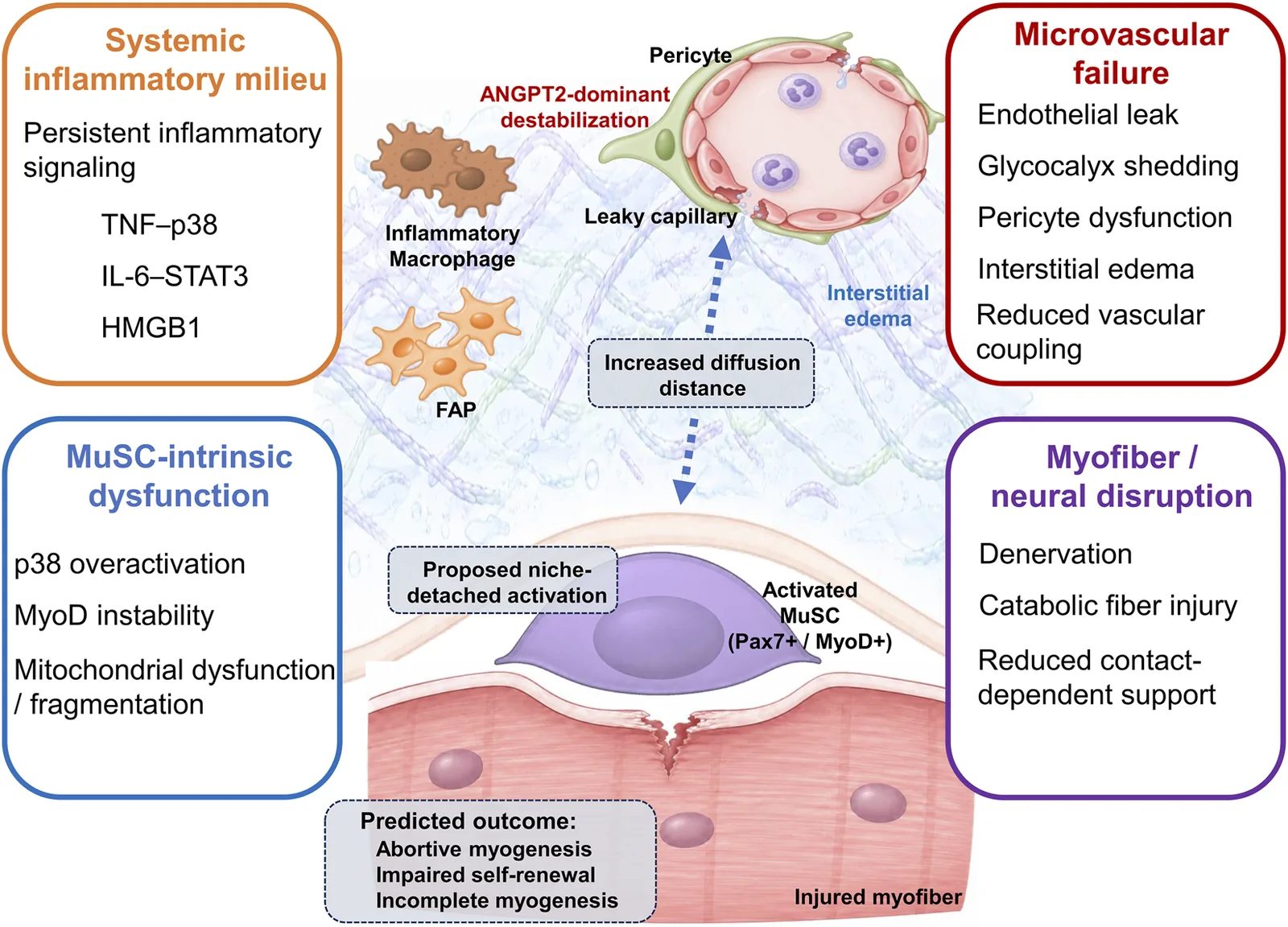
Acute disruption of the satellite cell niche during critical illness. Systemic inflammation, microvascular failure, neural and myofiber injury, and satellite cell-intrinsic dysfunction all act on the regenerative niche during critical illness. Endothelial barrier disruption, glycocalyx loss, Angiopoietin-2 (ANGPT2)-dominant signaling, and pericyte dysfunction promote interstitial edema. They may reduce vascular support to satellite cells, while myofiber injury and denervation diminish contact-dependent signals from the parent fiber. In parallel, excessive p38 signaling, mitochondrial dysfunction or fragmentation, and instability of the myogenic program may impair satellite cell function. We propose that activation under these conditions occurs in a poorly supportive niche, a state we term niche-detached activation, and that a cell activated in this setting may begin the myogenic program without completing it, a pattern we term abortive myogenesis. Dashed arrows and boxes indicate proposed relationships or outcomes rather than mechanisms established directly in human critical illness.

## 4 Mechanisms of satellite cell dysfunction

The satellite cell is not a passive recipient of niche-derived signals. Critical illness may alter the cell itself in ways that impair repair even if the niche is restored, so the two aspects are complementary: a damaged satellite cell within a damaged niche.

### 4.1 Excess p38 and impaired self-renewal

Much of what we know about cell-autonomous satellite cell failure comes from aging, where the parallels with critical illness are striking, even though the two are not the same. In aged muscle, satellite cells lose the ability to renew themselves through a defect intrinsic to the cell rather than to its environment, and the defect runs through excess p38α/β mitogen-activated protein kinase, whose elevated activity impairs self-renewal (Bernet et al., 2014). This link is functionally important: culturing aged satellite cells with a p38α/β inhibitor on a soft substrate that matches the elasticity of healthy muscle expands functional stem cells that, once transplanted, restore force to injured aged muscle (Cosgrove et al., 2014). In critical illness, p38 may mediate inflammatory effects on satellite cells, since tumor necrosis factor signals through p38 to the Pax7 locus (Section 3.2), so acute-illness cytokines may activate the same kinase shown to impair self-renewal in aging. In normal repair, asymmetric p38 activity directs one daughter toward commitment while the other retains stem-cell identity (Section 2.6). If p38 is held high, that balance is expected to tilt toward commitment and away from self-renewal. The cell’s progeny would then be biased toward differentiation and the pool would not be refilled. We consider this a mechanistic parallel rather than an established mechanism in muscle from critically ill patients, but it suggests a potential therapeutic target.

### 4.2 Weakened Wnt7a-driven expansion

Wnt7a-dependent expansion may also be impaired. Expansion of the satellite stem-cell pool depends on Wnt7a acting through Frizzled-7 and the planar cell polarity pathway to drive symmetric, pool-expanding divisions (Le Grand et al., 2009; Bentzinger et al., 2013). Fibronectin in the niche is required for efficient presentation of the Wnt7a signal (Bentzinger et al., 2013), and fibronectin is lost from the aged stem-cell niche (Lukjanenko et al., 2016). Critical illness is associated with systemic matrix remodeling, but whether fibronectin is specifically depleted from the satellite cell niche during critical illness is not known. If it is, Wnt7a-dependent expansion would be weakened at a time when regenerative demand is high. This possibility remains inferential, but it provides a second route by which self-renewal could fail: the stem-cell compartment may be unable to expand normally even when baseline satellite cell number is not yet reduced.

### 4.3 Mitochondrial and metabolic reprogramming

Mitochondrial findings in satellite cells vary across experimental models and clinical studies. A mouse model of sepsis caused persistent metabolic and mitochondrial abnormalities in satellite cells (Rocheteau et al., 2015). Cultured satellite cells from ten patients with ICU-acquired weakness had lower maximal respiratory capacity, ATP production, and glycolytic capacity, together with increased reactive oxygen species production and impaired complex II function (Genserová et al., 2024). In the SATELLITE cohort, overall bioenergetics and substrate preference were broadly preserved, while greater mitochondrial fragmentation was associated with lower proliferative capacity and muscle strength (Krajčová et al., 2026). These studies do not establish whether the differing findings reflect disease stage, patient characteristics, or experimental conditions. At the whole-muscle level, chronic weakness and mitochondrial dysfunction can persist after sepsis in mice even without sustained atrophy (Owen et al., 2019). Pharmacological protection of mitochondria can prevent chronic post-sepsis weakness in animals (Kingren et al., 2024), and urolithin A has improved long-term muscle and mitochondrial abnormalities in a sepsis model in which autophagy was disrupted (Pierre et al., 2025b). Persistent mitochondrial abnormalities have also been reported in muscle from survivors of critical COVID-19 (Mayer et al., 2024), while proposed links between cuproptosis and sepsis-acquired weakness remain preliminary (Yang et al., 2025).

These observations make it difficult to attribute the problem simply to an ATP shortage. A quiescent satellite cell relies on fatty-acid oxidation, undergoes a metabolic shift as it activates, and uses autophagy to meet the demands of that transition; mitochondrial content and dynamics also influence cell fate (Tang and Rando, 2014; Bhattacharya and Scimè, 2020). Disturbance of mitochondrial turnover could therefore impair regeneration through several routes, including altered redox state, accumulation of damaged organelles, or failure to make the metabolic transition required for myogenesis, even when bulk respiratory measurements remain relatively preserved (Chatre et al., 2017). In this setting, mitochondrial morphology and quality control may matter as much as overall respiratory capacity. Mitochondrial dysfunction should therefore be treated as a plausible component of satellite cell failure rather than a uniform or isolated lesion.

### 4.4 Satellite cell senescence

Cellular senescence is a stable state of cell-cycle arrest that differs from reversible satellite cell quiescence. In geriatric mice, derepression of p16INK4a promotes satellite cell senescence and impairs regeneration (Sousa-Victor et al., 2014). Loss of basal autophagy can also induce senescence through impaired proteostasis, mitochondrial dysfunction, and oxidative stress (García-Prat et al., 2016). Evidence linking these processes to critical illness is more limited. Chen et al. (2023) reported increased p53 and p21 expression and senescence-associated β-galactosidase staining in skeletal muscle from septic mice, with related changes in LPS-treated myoblasts. These observations do not establish persistent senescence of satellite cells in human survivors of critical illness. Distinguishing senescence from transient stress responses and reversible cell-cycle arrest requires cell-specific assessment over time, using multiple markers and functional measures.

### 4.5 FOXO, atrogin-1, and the instability of MyoD

A final potential cell-autonomous link ties the atrophy machinery to the regenerative one. In muscle fibers, insulin-like growth factor 1 signals through PI3-kinase and AKT to keep the FOXO factors phosphorylated and out of the nucleus. In satellite cells, FOXO3 helps promote return to quiescence during self-renewal (Section 2.3; Brunet et al., 1999; Gopinath et al., 2014). FOXO activity is therefore context-dependent: in muscle fibers, reduced IGF-1–AKT signaling permits nuclear FOXO factors to induce the atrophy ligases atrogin-1 and MuRF1 (Section 3.3; Sandri et al., 2004; Bodine and Baehr, 2014). For regeneration, the key connection is that atrogin-1, also called MAFbx, ubiquitinates MyoD and promotes its proteasomal degradation in cultured myogenic cells (Tintignac et al., 2005). The same catabolic program that promotes the loss of contractile protein could therefore also destabilize the myogenic regulatory factor a satellite cell needs to differentiate.

These cell-autonomous defects suggest a second working hypothesis, subject to the same caution as the first. Suppose a satellite cell activates, whether appropriately after injury or, as Section 3 implies, prematurely in response to the disrupted niche. The conditions it then encounters may be unfavorable both inside the cell and in the surrounding niche. Under this hypothesis, excess p38 signaling may redirect divisions away from self-renewal, and Wnt7a-dependent symmetric expansion may be weakened. Inside the cell, mitochondrial damage and impaired quality control may disturb the remodeling that accompanies myogenic progression (Section 4.3), senescence-like state changes may reduce the responsive pool, and the catabolic machinery that promotes muscle wasting may destabilize MyoD. An activated cell in this state may initiate the myogenic program but fail to complete it, neither replenishing the reserve pool nor producing functional new fibers. We call this abortive myogenesis (Figure 3). The model predicts that muscle from critically ill patients will contain activated, MyoD-positive satellite cells that fail to progress to fusion, together with poor replenishment of the reserve pool and a regenerative response that stalls midway rather than failing to start. The SATELLITE study provides an initial human observation consistent with this model. At the first biopsy, myogenic differentiation was reduced in culture, and the positive correlation between proliferation and fusion observed in controls was not detected, which the authors interpreted as disrupted coordination of the myogenic program rather than a uniform loss of myogenic capacity (Krajčová et al., 2026). This study has two important limitations. The day-7 fusion index did not differ significantly from controls, so the hypothesis as stated here, of a failure to complete myogenesis that persists long enough to matter for recovery, is not yet established. The assays were also performed on isolated cells in culture, and therefore assess cellular capacity under culture conditions rather than behavior within the niche. The observation shows that aspects of the prediction can be assessed using patient-derived cells. Whether this mechanism impairs satellite cell-mediated regeneration in ICU-AW remains to be established.

## 5 From acute injury to a state that persists

Neither a compromised niche nor a cell that is itself impaired explains, on its own, why the problem outlasts the illness by months or years. This section examines how cell-intrinsic defects and niche abnormalities may persist after acute injury. Much of the direct human evidence is still limited, so we draw on parallel conditions, disuse, chronic lung disease, aging, and long COVID, in which the same machinery has been studied over longer periods.

### 5.1 What the longitudinal human evidence shows

Human longitudinal evidence remains sparse at the satellite-cell level, but at the tissue level it is now substantial and no longer points simply to the depletion of the stem-cell pool. The pilot work discussed earlier found chronic wasting and impaired myogenic capacity months after the ICU stay, with reduced satellite cell content in patients who remained weak and atrophic (Dos Santos et al., 2016). The SATELLITE study reveals a different pattern: satellite cell number was preserved during the first week of critical illness and remained close to control values in the small group biopsied at day 180 (n = 4–6, depending on the assay), whereas proliferative capacity at day 180 appeared reduced and physical function was still impaired (Krajčová et al., 2026). This suggests that impaired proliferative capacity may persist even when the stem-cell pool is not numerically exhausted. In the earlier survivor cohort, network analysis of the muscle transcriptome identified modules enriched for regeneration and extracellular-matrix genes that can be linked to weakness and atrophy over time (Walsh et al., 2016). Larger and longer-term data have since become available. In a pre-planned secondary analysis of the EPaNIC trial, muscle from 115 former ICU patients was biopsied 5 years after critical illness and compared with muscle from 30 matched controls. After adjustment for age, sex, and body mass index, 234 transcripts were downregulated and 116 upregulated, with over-representation of pathways for mitochondrial energy metabolism, lipid metabolism, and collagen formation. Several of these were associated with the patients’ long-term muscle strength (Uzun Ayar et al., 2025). A companion analysis of the same cohort found that abnormal DNA methylation in muscle at 5 years correlated with these altered transcripts, most strongly among the fibrosis-related genes, and that such correlations were more frequent among transcripts linked to reduced strength (Uzun Ayar et al., 2026). Older age, in-ICU glucocorticoid exposure, and early parenteral nutrition were among the factors associated with more abnormal methylation. The imaging and biopsy observations noted in Section 1.2 point in the same direction, with fibrous and fatty remodeling replacing normal architecture (Mart et al., 2026). Survivors of critical COVID-19 provide a disease-specific analog: muscle biopsied roughly 9 months after discharge showed persistent mitochondrial abnormalities and reduced activity of respiratory complexes II and IV alongside fatigue and weakness (Mayer et al., 2024). These studies differ in size and design, but they support a chronic defect in muscle recovery without establishing a single mechanism.

### 5.2 A stiff, fibrotic matrix as a persistent barrier to regeneration

One reason an injury may fail to resolve is that the extracellular matrix can remain abnormal after injury. As described in Section 2.4, injury stiffens the matrix, and the stiff matrix keeps satellite cells activated through YAP and TAZ. Therefore, failure to soften the matrix would keep the cells in an activated state rather than allowing them to return to quiescence (Silver et al., 2021). The stiffness reflects not only how much collagen is present but how it is organized and cross-linked: covalent cross-linking stiffens collagen and slows its turnover, so that a fibrotic niche tends to perpetuate itself; even so, passive muscle stiffness reflects collagen content, fiber alignment, and higher-order architecture as well, and not cross-linking alone (Gillies and Lieber, 2011). The matrix can also redirect satellite cell fate: an aged or altered extracellular matrix can push satellite cells toward a fibrogenic program in culture, with increased expression of fibrogenic markers and reduced myogenicity (Stearns-Reider et al., 2017), while the loss of niche fibronectin weakens the signals that normally expand the pool (Lukjanenko et al., 2016). In muscle from critically ill patients, where recovery can give way to fibrosis and where the diaphragm in particular shows substantial replacement of muscle by fibrous tissue (Sections 1.2 and 2.8), it is plausible that the matrix itself becomes a lasting obstacle, holding satellite cells in an inappropriate state long after the original signals have gone. Direct evidence for this remodeling in post-ICU limb muscle remains limited; the proposal rests largely on matrix biology established in injury and aging models, together with the fibrosis documented in the ventilated diaphragm. The transcriptomic and methylation data discussed in Section 5.1 provide molecular insight: 5 years after critical illness, fibrosis-related transcripts remain abnormal and are most closely tied to abnormal DNA methylation (Uzun Ayar et al., 2025; Uzun Ayar et al., 2026). A structural memory encoded in the matrix and epigenetic changes in muscle tissue are not conflicting explanations, and either could help account for why the phenotype outlasts the signals that produced it. These bulk-tissue data do not identify the responsible cell types or establish causality.

### 5.3 A vascular niche that does not recover

A second potential mechanism involves the vascular niche. Because satellite cells depend on close proximity to capillaries for quiescence and survival (Section 2.3), anything that reduces the microvasculature or increases its distance from the cell would be expected to degrade the niche; relevant analogs come from disuse, chronic disease, and aging (McKenna and Fry, 2017). Two weeks of bed rest reduce satellite cell content alongside fiber atrophy in middle-aged adults (Arentson-Lantz et al., 2016), and capillary rarefaction occurs during bed rest, but is proportionally smaller than fiber atrophy (Hendrickse et al., 2022). Disuse atrophy is multidimensional, with myonuclei, satellite cell behavior, and several signaling pathways changing together (Brooks and Myburgh, 2014). Disuse alone does not always produce this. Disuse atrophy by itself, without an inflammatory or injurious component, may leave satellite cell content unchanged (Snijders et al., 2014), which suggests that inflammation and injury combined with disuse and vascular loss, and not inactivity by itself, compromise the regenerative compartment. Aging may exacerbate these changes, since older muscle regrows poorly and expands its myogenic precursors less effectively after immobilization (Suetta et al., 2013). The microcirculation is increasingly regarded as a determinant of muscle function and plasticity in its own right (Hendrickse and Degens, 2019), and the effects are reciprocal. Capillarization is associated with the fiber hypertrophy that resistance training can achieve in older men (Snijders et al., 2017), and in healthy aging the size of a fiber and its capillary supply stay tightly coupled (Barnouin et al., 2017). Chronic disease and aging appear to break that coupling. In chronic obstructive pulmonary disease, satellite cells lie at a greater distance from capillaries than in healthy controls (Betz et al., 2024), and aging muscle shows apoptosis of capillary endothelial cells, a loss of the vascular bed that is not simply secondary to fiber shrinkage (Wang et al., 2014). Whether critical illness uncouples the satellite cell from its capillary in the same way has not been shown directly in post-ICU muscle. Still, the parallel conditions make it plausible. Long COVID provides a further comparison, with persistent sublingual capillary rarefaction reported in affected patients (Osiaevi et al., 2023) and a growing body of work tying skeletal muscle abnormalities, including microvascular and mitochondrial changes, to post-exertional malaise (Appelman et al., 2024; Charlton et al., 2025); these are not direct evidence for post-ICU muscle, but they offer a close comparison for chronic post-inflammatory muscle dysfunction, and an overlap with myalgic encephalomyelitis and chronic fatigue syndrome has been noted (Scheibenbogen and Wirth, 2025). Two findings argue against treating this as irreversible. Capillary rarefaction is not necessarily permanent, since overload-driven angiogenesis can restore muscle performance (Tickle et al., 2020), and transplanting pericytes improves muscle recovery after immobilization (Munroe et al., 2019). The vascular part of the niche is therefore a likely site of lasting damage and a potential point of repair.

### 5.4 Overlap with the aged niche

A stiff fibrotic matrix, a rarefied vasculature, persistent inflammation, and senescence-related changes also occur in aged muscle. The aging literature shows that both the niche and the satellite cell influence regenerative capacity. Exposing old satellite cells to a young systemic environment restores much of their function, and the converse also holds, showing that blood-borne factors, not only the local tissue, carry the age signal (Conboy et al., 2005). Restoring Notch signaling rejuvenates aged muscle regeneration (Conboy et al., 2003), whereas rising Wnt signaling with age pushes satellite cells toward a fibrogenic fate and increases fibrosis (Brack et al., 2007). The aged niche actively disrupts satellite cell quiescence, in part through fibroblast growth factor signaling that promotes cell-cycle entry in resting cells and slowly exhausts the pool (Chakkalakal et al., 2012), with TGF-β1 and Wnt among the systemic signals that drive the decline (Carlson et al., 2009). Satellite cell loss in aging is especially notable in type II fibers (Verdijk et al., 2007).

Chronological age measures elapsed time, whereas biological aging concerns changes in function and resilience that vary among individuals of the same age; neither age in years nor a frailty score directly establishes the biological state of satellite cells or their niche. Critical illness and chronic disease share several features with aging, including inflammation, fibrosis, and mitochondrial abnormalities, but this overlap does not establish accelerated cellular aging, which is distinct from the cellular senescence discussed in Section 4.4. Critical illness may expose pre-existing impairment, cause additional injury, or both. In the SATELLITE cohort, older age was associated with lower early satellite cell proliferative capacity (Krajčová et al., 2026). The younger control group and the absence of pre-illness measurements make it difficult to separate age-related impairment from changes caused by the acute illness. Future studies should record chronological age, premorbid function, frailty, and chronic disease, and assess relevant cellular abnormalities where tissue is available. Statistical adjustment can address confounding by age. Prespecified age-by-illness or age-by-treatment interaction analyses can test whether associations or treatment effects vary with age. Analyses within age strata may aid interpretation when sample sizes are sufficient, but should complement appropriate adjustment.

### 5.5 Variable outcomes of rehabilitation

If the niche is part of the problem, exercise directed at the muscle fiber and its satellite cells may not be sufficient on its own, and clinical trials have not shown a consistent long-term benefit from simply increasing rehabilitation intensity. Early active mobilization during mechanical ventilation did not increase days alive and out of hospital and was associated with more adverse events (Hodgson et al., 2022); an intensive physical therapy program after acute respiratory failure did not improve long-term physical function (Moss et al., 2016). These results are usually interpreted in terms of dose, timing, and case mix, and they do not show that the muscle tissue itself is unresponsive. Mechanical loading depends on an intact matrix, adequate perfusion, and a regenerative compartment able to respond. A stiff or disorganized matrix may distort mechanical signaling, a rarefied microvasculature may limit oxygen and nutrient delivery, and a poorly responsive satellite cell compartment, whether or not numerically depleted, may contribute little to new tissue formation. The small blood-flow-restricted exercise study discussed earlier is consistent with this view: the regimen expanded the satellite cell pool in healthy controls but not in the two survivors with persistent atrophy, whereas the survivor whose muscle mass had recovered responded normally (Mathur et al., 2025). This does not prove niche failure as the cause of rehabilitation resistance. Still, it suggests that tissue state may help determine whether a loading stimulus can be converted into regeneration.

The persistence described in this section may reflect more than slow healing. We propose, as a third and final hypothesis, that critical illness may, in some patients, drive the niche into a self-sustaining, non-permissive state that holds satellite cells out of productive repair, a state we call niche locking (Figure 4). Its proposed components are those described above: a fibrotic, stiff matrix that sustains abnormal mechanotransduction; a rarefied microvasculature that provides less trophic support and is spatially uncoupled from satellite cells; unresolved low-grade inflammation; and senescent or senescence-prone cells. Each could reinforce the others after the original insult has resolved. The three hypotheses can also be arranged as one possible trajectory, running from niche-detached activation in the acute phase, through abortive myogenesis as cells fail to complete repair, to niche locking as the tissue settles into a non-regenerative state. They need not occur in every patient or in a fixed order. The model makes a therapeutic prediction that differs from a simple atrophy model: if a locked niche exists, restoring regeneration may require correcting the niche itself through revascularization, matrix normalization, control of persistent inflammation, or other approaches, rather than stimulating the satellite cell alone.

**FIGURE 4:**
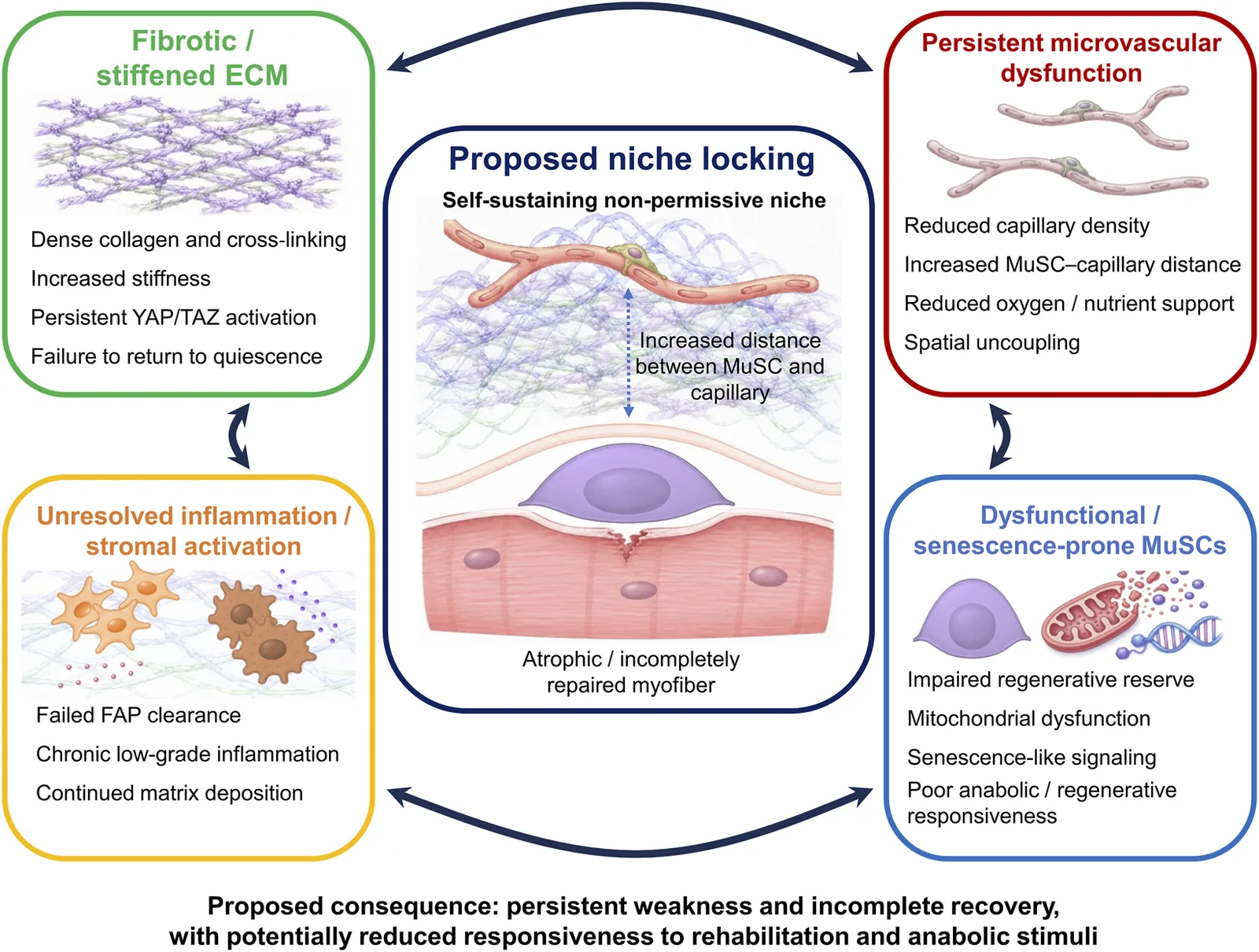
Proposed niche locking after critical illness. Persistent abnormalities in the extracellular matrix (ECM), microvasculature, inflammatory and stromal compartments, and muscle satellite cells (MuSCs) may reinforce one another after the acute phase of critical illness, establishing a self-sustaining, non-permissive regenerative niche. Fibrotic matrix accumulation and cross-linking may increase tissue stiffness and sustain abnormal mechanotransduction. Incomplete microvascular recovery may increase the distance between satellite cells and capillaries and reduce vascular trophic support. Persistent inflammation or stromal activation, including incomplete clearance of fibro-adipogenic progenitors (FAPs), could promote continued matrix deposition. At the same time, satellite cells may retain impaired regenerative reserve, mitochondrial abnormalities, and senescence-like features. We call this proposed persistent state niche locking. Such a niche could limit muscle’s ability to convert rehabilitation or anabolic stimuli into effective regeneration, thereby contributing to persistent weakness and incomplete recovery. This model is hypothetical; several components, particularly persistent MuSC–capillary uncoupling and satellite cell senescence, have not been demonstrated directly in human survivors of critical illness. Curved arrows indicate proposed reinforcing interactions among niche compartments.

## 6 Measurement, treatment, and what to study next

### 6.1 Measuring the satellite cell and its niche in patients

A practical limitation concerns measurement. The state of the satellite cell and its niche cannot be measured easily at the bedside. The biomarkers available for ICU-AW are largely circulating markers of inflammation and nerve or vascular injury. Although several have predictive or prognostic value, none directly reports the state of the satellite cell compartment or its niche (Song et al., 2025). Longitudinal blood profiling is beginning to show how these markers change over the course of illness (Klawitter et al., 2025). Still, a circulating signal does not directly measure stem-cell behavior in tissue. Ultrasound measures muscle size and architecture rather than regenerative function (Umbrello et al., 2024). Functional challenge offers another route of assessment: the blood-flow-restricted exercise study in long-term survivors asked whether the satellite cell compartment could still respond to an anabolic stimulus (Mathur et al., 2025). Serial biopsy is difficult but feasible. The SATELLITE study obtained vastus lateralis samples within 72 h of ICU admission, at day 7, and again at day 180 in a small survivor subgroup (Krajčová et al., 2026). Serial measurement of the cell together with its niche remains incomplete, particularly for measures such as capillary density and satellite cell-to-capillary distance, matrix composition and stiffness, inflammatory and stromal cell states, and markers of senescence or persistent activation.

### 6.2 Single-cell, spatial, and preclinical tools

Methods are now available to profile transcriptomic and epigenetic changes at single-cell resolution. Single-cell RNA sequencing has resolved the heterogeneity of human satellite cells (Barruet et al., 2020), mapped the satellite cell hierarchy and the signals cells exchange during repair (De Micheli et al., 2020), and, through large-scale integration, captured the transitional states that cells pass through as they regenerate (McKellar et al., 2021); profiling of chromatin accessibility has defined a self-renewing satellite cell state at the level of gene regulation (Okafor et al., 2023). Several of these studies examine the niche interactions this review has emphasized. Single-cell and spatial transcriptomics together identified a macrophage population tied to fibrosis (Coulis et al., 2023), uncovered a satellite cell–FAP signal carried by FGF7 (Ma et al., 2024), and showed that satellite cells differ among themselves in the inflammatory chemokines they express (Andre et al., 2023). Spatial methods are particularly useful here, because they preserve the spatial organization of gene expression without requiring complete tissue dissociation (Virtanen et al., 2025). The chief obstacle to applying these tools in critical illness is the model: preclinical ICU-AW models vary, and none fully reproduces the human syndrome (Yu et al., 2024); human ICU biopsies remain scarce and longitudinal sampling is difficult. Testing the three hypotheses we have set out will likely require combining opportunistic clinical biopsies and carefully phenotyped survivor cohorts with better-standardized animal models, asking, for instance, whether activated satellite cells in muscle from critically ill patients stall before fusion, and whether the vascular and matrix components of their niche are still disrupted when they stall.

### 6.3 Therapeutic directions

Therapeutic strategies may need to restore the cell’s capacity to respond and the niche’s capacity to support it. These approaches remain experimental rather than established treatments for ICU-AW; most of the supporting evidence comes from preclinical sepsis models, aging biology, sarcopenia, COPD, or early experimental work in survivors. Mitochondrial quality control is one potential target. In cultured satellite cells from patients with ICU-acquired weakness, β-hydroxybutyrate restored complex II function and reduced reactive oxygen species production, without a significant effect on overall mitochondrial function (Genserová et al., 2024). These results do not establish clinical efficacy in ICU-AW. Urolithin A, a metabolite that promotes mitophagy and mitochondrial turnover, improved muscle endurance or strength and markers of mitochondrial health in randomized trials in middle-aged and older adults (Liu et al., 2022; Singh et al., 2022), and it improved long-term muscle and mitochondrial abnormalities in a sepsis model (Pierre et al., 2025b); pharmacological mitochondrial protection also prevented post-sepsis weakness in animals (Kingren et al., 2024). Anabolic approaches have been tried directly in survivors: the GAINS pilot combined an anabolic steroid with nutrition and exercise (Anstey et al., 2022), while activin type II receptor blockade with bimagrumab increased muscle mass in sarcopenia and COPD without a consistent gain in function (Polkey et al., 2019; Rooks et al., 2020). Targeting the satellite cell itself is plausible in principle, since dampening p38 restores self-renewal to aged satellite cells (Bernet et al., 2014; Cosgrove et al., 2014), but systemic p38 inhibition would require caution because the pathway also participates in inflammation, stress responses, and normal myogenic progression.

Transient expression of Yamanaka factors has been examined as an experimental strategy to promote muscle regeneration. In young mice, induction of Oct4, Sox2, Klf4, and c-Myc in myofibers before cardiotoxin injury accelerated regeneration. This effect involved increased myofiber p21 expression, reduced Wnt4 expression, and satellite cell activation. Induction in satellite cells, or in myofibers after injury, did not improve regeneration under the tested conditions (Wang et al., 2021). These results support further investigation of niche modification but do not establish reversal of satellite cell aging or efficacy in ICU-AW. Application to critical illness would require evidence of benefit after established injury, recovery of muscle function, and control of tissue targeting and expression duration, with assessment of tumor risk.

The approaches that most directly target niche locking are less mature: restoring the microvasculature, normalizing the matrix, and clearing senescent or senescence-like cells, the last requiring evidence that such cells accumulate in post-ICU muscle. Cell-based strategies are also experimental; mesenchymal stromal cells restored satellite cell mitochondrial function and regenerative capacity in a sepsis model (Rocheteau et al., 2015). Timing likely matters as well. Trials of increased rehabilitation intensity have not shown consistent benefits (Moss et al., 2016; Hodgson et al., 2022), raising the possibility that regenerative therapies and loading may need to match the state of the muscle and its niche.

### 6.4 Outstanding questions

The discussion above identifies several questions that current tools can begin to address; they fall naturally under the three working hypotheses. The first concerns activation timing. The SATELLITE study has begun to define the early human response: satellite cell number was preserved over the first week, while myogenic differentiation was already impaired in culture using cells obtained at the initial biopsy (Krajčová et al., 2026). It is still unknown whether satellite cells leave quiescence prematurely *in vivo* during critical illness. At the same time, the surrounding niche may still be disrupted, or the contact-dependent signals that normally maintain quiescence may be lost or merely altered. Answering that question will require early serial sampling paired with cell-state markers and measurements of the surrounding vascular, matrix, and interstitial compartments. A related question is whether the close physical relationship between satellite cells and capillaries is durably lost in survivors and whether that loss tracks with recovery.

The second concerns whether activation, once it occurs, is productive. The new longitudinal data narrow the question: differentiation can be impaired early, and proliferative capacity may remain reduced months later even when satellite cell number remains relatively preserved (Krajčová et al., 2026). What remains unknown is where the program fails *in vivo*: activation, maintenance of MyoD, induction of Myogenin, fusion, addition of new myonuclei, or maturation of the repaired fiber. Defining that point would identify the stage at which myogenesis is impaired. It would help determine whether mitochondrial abnormalities are a cause of the arrest, a concurrent consequence of critical illness, or both.

The third concerns whether the niche becomes self-sustaining. It remains to be shown whether the matrix of survivor muscle carries a fibrotic, stiff signature able to hold satellite cells in a fibrogenic or persistently activated state, and whether senescent or senescence-like cells accumulate at all in post-ICU muscle. No senolytic strategy has met this prerequisite. The question that matters most for treatment is whether niche locking is reversible and, if so, which element, the matrix, the microvasculature, inflammation, metabolism, or senescence, must be corrected first for regeneration to resume.

All three share the question of causation. The critical-illness milieu combines inflammation, glucocorticoid exposure, hyperglycemia, immobilization, and denervation, and it is not known which of these impair satellite cell-mediated regeneration on their own and which act only in combination. Nor is it clear how much of the defect is intrinsic to the cell and how much is imposed by its surroundings, a distinction that serum-transfer and cross-environment transplantation experiments are suited to draw, and one that bears directly on whether therapy should target the cell, the niche, or both.

## 7 Conclusion

Weakness after critical illness has long been understood mainly as the consequence of acute muscle breakdown, but breakdown alone does not fully explain why weakness persists. We have argued that persistent weakness may also reflect a failure of regeneration and that the relevant unit is not the satellite cell alone but the satellite cell together with its niche. The niche is multicellular and spatially organized, built from the parent fiber, microvasculature and pericytes, extracellular matrix, and resident immune and interstitial cells. Critical illness can disturb each of these compartments while also altering the satellite cell from within. Recent human data argue against a simple model in which early loss of satellite cell number explains the regenerative problem; impaired differentiation and reduced proliferative capacity in culture can occur despite relative preservation of the cell pool. We have used three working hypotheses to organize this evidence: satellite cells may activate in a niche that can no longer support orderly repair, they may begin myogenesis without completing it, and in some survivors the niche may settle into a state that continues to resist regeneration. These hypotheses may overlap or occur in different temporal orders, and each remains to be tested directly. Single-cell, spatial, and longitudinal tissue studies now make that testing possible. Future studies should examine why muscle regeneration remains impaired in some survivors.

## References

[B1] Abou-Khalil R. Le Grand F. Pallafacchina G. Valable S. Authier F. J. Rudnicki M. A. (2009). Autocrine and paracrine angiopoietin 1/Tie-2 signaling promotes muscle satellite cell self-renewal. Cell. Stem Cell. 5, 298–309. 10.1016/j.stem.2009.06.001 19733541 PMC4592285

[B2] Alexander K. A. Tseng H. W. Lao H. W. Girard D. Barbier V. Ungerer J. P. J. (2024). A glucocorticoid spike derails muscle repair to heterotopic ossification after spinal cord injury. Cell. Rep. Med. 5, 101849. 10.1016/j.xcrm.2024.101849 39657663 PMC11722129

[B3] Almada A. E. Wagers A. J. (2016). Molecular circuitry of stem cell fate in skeletal muscle regeneration, ageing and disease. Nat. Rev. Mol. Cell. Biol. 17, 267–279. 10.1038/nrm.2016.7 26956195 PMC4918817

[B4] Alvarez A. M. DeOcesano-Pereira C. Teixeira C. Moreira V. (2020). IL-1β and TNF-α modulation of proliferated and committed myoblasts: IL-6 and COX-2-derived prostaglandins as key actors in the mechanisms involved. Cells 9, 2005. 10.3390/cells9092005 32882817 PMC7564831

[B5] Andre A. B. Rees K. P. O'Connor S. Severson G. W. Newbern J. M. Wilson-Rawls J. (2023). Single cell analysis reveals satellite cell heterogeneity for proinflammatory chemokine expression. Front. Cell. Dev. Biol. 11, 1084068. 10.3389/fcell.2023.1084068 PMC10083252 37051469

[B6] Anstey M. H. Rauniyar R. Fitzclarence E. Tran N. Osnain E. Mammana B. (2022). Muscle growth and anabolism in intensive care survivors (GAINS) trial: a pilot randomised controlled trial. Acute Crit. Care 37, 295–302. 10.4266/acc.2021.01767 PMC9475160 35791659

[B7] Appelman B. Charlton B. T. Goulding R. P. Kerkhoff T. J. Breedveld E. A. Noort W. (2024). Muscle abnormalities worsen after post-exertional malaise in long COVID. Nat. Commun. 15, 17. 10.1038/s41467-023-44432-3 PMC10766651 38177128

[B8] Arentson-Lantz E. J. English K. L. Paddon-Jones D. Fry C. S. (2016). Fourteen days of bed rest induces a decline in satellite cell content and robust atrophy of skeletal muscle fibers in middle-aged adults. J. Appl. Physiol. (1985) 120, 965–975. 10.1152/japplphysiol.00799.2015 26796754 PMC4835912

[B9] Arnold L. Henry A. Poron F. Baba-Amer Y. van Rooijen N. Plonquet A. (2007). Inflammatory monocytes recruited after skeletal muscle injury switch into antiinflammatory macrophages to support myogenesis. J. Exp. Med. 204, 1057–1069. 10.1084/jem.20070075 17485518 PMC2118577

[B10] Asakura A. Rudnicki M. A. (2002). “Cellular and molecular mechanisms regulating skeletal muscle development,” in Mouse Development. Editors Rossant J. Tam P. P. L. (Orlando, FL: Academic Press), 253–278. 10.1016/B978-012597951-1/50016-0

[B11] Asakura A. Tapscott S. J. (1998). Apoptosis of epaxial myotome in Danforth's short-tail (Sd) mice in somites that form following notochord degeneration. Dev. Biol. 203, 276–289. 10.1006/dbio.1998.9050 9808779

[B12] Asakura A. Komaki M. Rudnicki M. A. (2001). Muscle satellite cells are multipotential stem cells that exhibit myogenic, osteogenic, and adipogenic differentiation. Differentiation 68, 245–253. 10.1046/j.1432-0436.2001.680412.x 11776477

[B13] Asakura A. Seale P. Girgis-Gabardo A. Rudnicki M. A. (2002b). Myogenic specification of side population cells in skeletal muscle. J. Cell. Biol. 159, 123–134. 10.1083/jcb.200202092 12379804 PMC2173497

[B14] Azzag K. Gransee H. M. Magli A. Yamashita A. M. S. Tungtur S. Ahlquist A. (2024). Enhanced diaphragm muscle function upon satellite cell transplantation in dystrophic mice. Int. J. Mol. Sci. 25, 2503. 10.3390/ijms25052503 38473751 PMC10931593

[B15] Baghdadi M. B. Castel D. Machado L. Fukada S. I. Birk D. E. Relaix F. (2018). Reciprocal signalling by Notch-collagen V-CALCR retains muscle stem cells in their niche. Nature 557, 714–718. 10.1038/s41586-018-0144-9 29795344 PMC5985950

[B16] Baht G. S. Bareja A. Lee D. E. Rao R. R. Huang R. Huebner J. L. (2020). Meteorin-like facilitates skeletal muscle repair through a STAT3/IGF-1 mechanism. Nat. Metab. 2, 278–289. 10.1038/s42255-020-0184-y 32694780 PMC7504545

[B17] Barnouin Y. McPhee J. S. Butler-Browne G. Bosutti A. De Vito G. Jones D. A. (2017). Coupling between skeletal muscle fiber size and capillarization is maintained during healthy aging. J. Cachexia Sarcopenia Muscle 8, 647–659. 10.1002/jcsm.12194 28382740 PMC5566646

[B18] Barruet E. Garcia S. M. Striedinger K. Wu J. Lee S. Byrnes L. (2020). Functionally heterogeneous human satellite cells identified by single cell RNA sequencing. eLife 9, e51576. 10.7554/eLife.51576 32234209 PMC7164960

[B19] Bentzinger C. F. Wang Y. X. Rudnicki M. A. (2012). Building muscle: molecular regulation of myogenesis. Cold Spring Harb. Perspect. Biol. 4, a008342. 10.1101/cshperspect.a008342 22300977 PMC3281568

[B20] Bentzinger C. F. Wang Y. X. von Maltzahn J. Soleimani V. D. Yin H. Rudnicki M. A. (2013). Fibronectin regulates Wnt7a signaling and satellite cell expansion. Cell. Stem Cell. 12, 75–87. 10.1016/j.stem.2012.09.015 23290138 PMC3539137

[B21] Bernet J. D. Doles J. D. Hall J. K. Kelly Tanaka K. Carter T. A. Olwin B. B. (2014). p38 MAPK signaling underlies a cell-autonomous loss of stem cell self-renewal in skeletal muscle of aged mice. Nat. Med. 20, 265–271. 10.1038/nm.3465 24531379 PMC4070883

[B22] Betz M. W. De Brandt J. Aussieker T. Monsegue A. P. Houtvast D. C. J. Gehlert S. (2024). Muscle fibre satellite cells are located at a greater distance from capillaries in patients with COPD compared with healthy controls. ERJ Open Res. 10, 00203–02024. 10.1183/23120541.00203-2024 39351378 PMC11440426

[B23] Bhattacharya D. Scimè A. (2020). Mitochondrial function in muscle stem cell fates. Front. Cell. Dev. Biol. 8, 480. 10.3389/fcell.2020.00480 32612995 PMC7308489

[B24] Birbrair A. Zhang T. Wang Z. M. Messi M. L. Enikolopov G. N. Mintz A. (2013). Skeletal muscle pericyte subtypes differ in their differentiation potential. Stem Cell. Res. 10, 67–84. 10.1016/j.scr.2012.09.003 23128780 PMC3781014

[B25] Bjornson C. R. Cheung T. H. Liu L. Tripathi P. V. Steeper K. M. Rando T. A. (2012). Notch signaling is necessary to maintain quiescence in adult muscle stem cells. Stem Cells 30, 232–242. 10.1002/stem.773 22045613 PMC3384696

[B26] Bodine S. C. Baehr L. M. (2014). Skeletal muscle atrophy and the E3 ubiquitin ligases MuRF1 and MAFbx/atrogin-1. Am. J. Physiol. Endocrinol. Metab. 307, E469–E484. 10.1152/ajpendo.00204.2014 25096180 PMC4166716

[B27] Boutet S. C. Cheung T. H. Quach N. L. Liu L. Prescott S. L. Edalati A. (2012). Alternative polyadenylation mediates microRNA regulation of muscle stem cell function. Cell. Stem Cell. 10, 327–336. 10.1016/j.stem.2012.01.017 22385659 PMC3306803

[B28] Brack A. S. Conboy M. J. Roy S. Lee M. Kuo C. J. Keller C. (2007). Increased Wnt signaling during aging alters muscle stem cell fate and increases fibrosis. Science 317, 807–810. 10.1126/science.1144090 17690295

[B29] Braun T. P. Marks D. L. (2015). The regulation of muscle mass by endogenous glucocorticoids. Front. Physiol. 6, 12. 10.3389/fphys.2015.00012 25691871 PMC4315033

[B30] Brooks N. E. Myburgh K. H. (2014). Skeletal muscle wasting with disuse atrophy is multi-dimensional: the response and interaction of myonuclei, satellite cells and signaling pathways. Front. Physiol. 5, 99. 10.3389/fphys.2014.00099 24672488 PMC3955994

[B31] Brunet A. Bonni A. Zigmond M. J. Lin M. Z. Juo P. Hu L. S. (1999). AKT promotes cell survival by phosphorylating and inhibiting a forkhead transcription factor. Cell. 96, 857–868. 10.1016/S0092-8674(00)80595-4 10102273

[B32] Burzyn D. Kuswanto W. Kolodin D. Shadrach J. L. Cerletti M. Jang Y. (2013). A special population of regulatory t cells potentiates muscle repair. Cell. 155, 1282–1295. 10.1016/j.cell.2013.10.054 24315098 PMC3894749

[B33] Cao Y. Y. Wang Z. Yu T. Zhang Y. Wang Z. H. Lu Z. M. (2021). Sepsis induces muscle atrophy by inhibiting proliferation and promoting apoptosis via PLK1-AKT signalling. J. Cell. Mol. Med. 25, 9724–9739. 10.1111/jcmm.16921 PMC8505846 34514712

[B34] Carlson M. E. Conboy M. J. Hsu M. Barchas L. Jeong J. Agrawal A. (2009). Relative roles of TGF-beta1 and Wnt in the systemic regulation and aging of satellite cell responses. Aging Cell. 8, 676–689. 10.1111/j.1474-9726.2009.00517.x 19732043 PMC2783265

[B35] Casaer M. P. Mesotten D. Hermans G. Wouters P. J. Schetz M. Meyfroidt G. (2011). Early versus late parenteral nutrition in critically ill adults. N. Engl. J. Med. 365, 506–517. 10.1056/NEJMoa1102662 21714640

[B36] Castillero E. Alamdari N. Aversa Z. Gurav A. Hasselgren P. O. (2013a). PPARβ/δ regulates glucocorticoid- and sepsis-induced FoxO1 activation and muscle wasting. PLoS One 8, e59726. 10.1371/journal.pone.0059726 23555761 PMC3605288

[B37] Castillero E. Alamdari N. Lecker S. H. Hasselgren P. O. (2013b). Suppression of atrogin-1 and MuRF1 prevents dexamethasone-induced atrophy of cultured myotubes. Metabolism 62, 1495–1502. 10.1016/j.metabol.2013.05.018 23866982

[B38] Chadda K. R. Puthucheary Z. (2024a). Persistent inflammation, immunosuppression, and catabolism syndrome (PICS): a review of definitions, potential therapies, and research priorities. Br. J. Anaesth. 132, 507–518. 10.1016/j.bja.2023.11.052 PMC10870139 38177003

[B39] Chadda K. R. Blakey E. E. Davies T. W. Puthucheary Z. (2024b). Risk factors, biomarkers, and mechanisms for persistent inflammation, immunosuppression, and catabolism syndrome (PICS): a systematic review and meta-analysis. Br. J. Anaesth. 133, 538–549. 10.1016/j.bja.2024.03.038 PMC11347813 38688799

[B40] Chakkalakal J. V. Jones K. M. Basson M. A. Brack A. S. (2012). The aged niche disrupts muscle stem cell quiescence. Nature 490, 355–360. 10.1038/nature11438 23023126 PMC3605795

[B41] Charlton B. T. Goulding R. P. Jaspers R. T. Appelman B. van Vugt M. Wüst R. C. I. (2025). Skeletal muscle adaptations and post-exertional malaise in long COVID. Trends Endocrinol. Metab. 36, 614–622. 10.1016/j.tem.2024.11.008 39694730

[B42] Chatre L. Verdonk F. Rocheteau P. Crochemore C. Chrétien F. Ricchetti M. (2017). A novel paradigm links mitochondrial dysfunction with muscle stem cell impairment in sepsis. Biochim. Biophys. Acta Mol. Basis Dis. 1863, 2546–2553. 10.1016/j.bbadis.2017.04.019 28456665

[B43] Chen J. Huang M. (2024). Intensive care unit-acquired weakness: recent insights. J. Intensive Med. 4, 73–80. 10.1016/j.jointm.2023.07.002 PMC10800771 38263973

[B44] Chen J. Chen X. Y. Cong X. X. Wang S. Xu S. B. Sun Y. T. (2023). Cellular senescence implicated in sepsis-induced muscle weakness and ameliorated with metformin. Shock 59, 646–656. 10.1097/SHK.0000000000002086 36719431

[B45] Cheng M. Nguyen M. H. Fantuzzi G. Koh T. J. (2008). Endogenous interferon-gamma is required for efficient skeletal muscle regeneration. Am. J. Physiol. Cell. Physiol. 294, C1183–C1191. 10.1152/ajpcell.00568.2007 18353892

[B46] Chi Y. Yu S. Yin J. Liu D. Zhuo M. Li X. (2024). Role of angiopoietin/Tie2 system in sepsis: a potential therapeutic target. Clin. Appl. Thromb. Hemost. 30, 10760296241238010. 10.1177/10760296241238010 PMC10921858 38449088

[B47] Christov C. Chrétien F. Abou-Khalil R. Bassez G. Vallet G. Authier F. J. (2007). Muscle satellite cells and endothelial cells: close neighbors and privileged partners. Mol. Biol. Cell. 18, 1397–1409. 10.1091/mbc.e06-08-0693 17287398 PMC1838982

[B48] Chuang Y. C. Shiu S. I. Lee Y. C. Tsai Y. L. Cheng Y. Y. (2025). Prevalence and risk factors of intensive care unit-acquired weakness in patients with COVID-19: a systematic review and meta-analysis. J. Intensive Care Med. 40, 1042–1051. 10.1177/08850666241268437 39140376

[B49] Clarke B. A. Drujan D. Willis M. S. Murphy L. O. Corpina R. A. Burova E. (2007). The E3 ligase MuRF1 degrades myosin heavy chain protein in dexamethasone-treated skeletal muscle. Cell. Metab. 6, 376–385. 10.1016/j.cmet.2007.09.009 17983583

[B50] Conboy I. M. Conboy M. J. Smythe G. M. Rando T. A. (2003). Notch-mediated restoration of regenerative potential to aged muscle. Science 302, 1575–1577. 10.1126/science.1087573 14645852

[B51] Conboy I. M. Conboy M. J. Wagers A. J. Girma E. R. Weissman I. L. Rando T. A. (2005). Rejuvenation of aged progenitor cells by exposure to a young systemic environment. Nature 433, 760–764. 10.1038/nature03260 15716955

[B52] Cosgrove B. D. Gilbert P. M. Porpiglia E. Mourkioti F. Lee S. P. Corbel S. Y. (2014). Rejuvenation of the muscle stem cell population restores strength to injured aged muscles. Nat. Med. 20, 255–264. 10.1038/nm.3464 24531378 PMC3949152

[B53] Coulis G. Jaime D. Guerrero-Juarez C. Kastenschmidt J. M. Farahat P. K. Nguyen Q. (2023). Single-cell and spatial transcriptomics identify a macrophage population associated with skeletal muscle fibrosis. Sci. Adv. 9, eadd9984. 10.1126/sciadv.add9984 PMC10328414 37418531

[B54] D'Souza D. M. Zhou S. Rebalka I. A. MacDonald B. Moradi J. Krause M. P. (2016). Decreased satellite cell number and function in humans and mice with type 1 diabetes is the result of altered notch signaling. Diabetes 65, 3053–3061. 10.2337/db15-1577 27335233

[B55] De Micheli A. J. Laurilliard E. J. Heinke C. L. Ravichandran H. Fraczek P. Soueid-Baumgarten S. (2020). Single-cell analysis of the muscle stem cell hierarchy identifies heterotypic communication signals involved in skeletal muscle regeneration. Cell. Rep. 30, 3583–3595.e5. 10.1016/j.celrep.2020.02.067 PMC7091476 32160558

[B56] Dejana E. Orsenigo F. Lampugnani M. G. (2008). The role of adherens junctions and VE-cadherin in the control of vascular permeability. J. Cell. Sci. 121, 2115–2122. 10.1242/jcs.017897 18565824

[B57] Dellavalle A. Maroli G. Covarello D. Azzoni E. Innocenzi A. Perani L. (2011). Pericytes resident in postnatal skeletal muscle differentiate into muscle fibres and generate satellite cells. Nat. Commun. 2, 499. 10.1038/ncomms1508 21988915

[B58] Derde S. Vanhorebeek I. Güiza F. Derese I. Gunst J. Fahrenkrog B. (2012). Early parenteral nutrition evokes a phenotype of autophagy deficiency in liver and skeletal muscle of critically ill rabbits. Endocrinology 153, 2267–2276. 10.1210/en.2011-2068 22396453

[B59] Ding J. Li Q. Liang F. Bhattarai S. Liu Q. (2021). Mechanical ventilation regulated the differentiation and proliferation of diaphragm satellite cells. Front. Biosci. Landmark Ed. 26, 1013–1018. 10.52586/5005 34856749

[B60] Dinglas V. D. Aronson Friedman L. Colantuoni E. Mendez-Tellez P. A. Shanholtz C. B. Ciesla N. D. (2017). Muscle weakness and 5-year survival in acute respiratory distress syndrome survivors. Crit. Care Med. 45, 446–453. 10.1097/CCM.0000000000002208 28067712 PMC5315580

[B61] Dong Y. Pan J. S. Zhang L. (2013). Myostatin suppression of Akirin1 mediates glucocorticoid-induced satellite cell dysfunction. PLoS One 8, e58554. 10.1371/journal.pone.0058554 23516508 PMC3596298

[B62] Dort J. Fabre P. Molina T. Dumont N. A. (2019). Macrophages are key regulators of stem cells during skeletal muscle regeneration and diseases. Stem Cells Int. 2019, 4761427. 10.1155/2019/4761427 31396285 PMC6664695

[B63] Dos Santos C. Hussain S. N. Mathur S. Picard M. Herridge M. Correa J. (2016). Mechanisms of chronic muscle wasting and dysfunction after an intensive care unit stay: a pilot study. Am. J. Respir. Crit. Care Med. 194, 821–830. 10.1164/rccm.201512-2344OC 27058306

[B64] Doyle A. Zhang G. Abdel Fattah E. A. Eissa N. T. Li Y. P. (2011). Toll-like receptor 4 mediates lipopolysaccharide-induced muscle catabolism via coordinate activation of ubiquitin-proteasome and autophagy-lysosome pathways. FASEB J. 25, 99–110. 10.1096/fj.10-164152 20826541 PMC3005430

[B65] Dres M. Goligher E. C. Heunks L. M. A. Brochard L. J. (2017). Critical illness-associated diaphragm weakness. Intensive Care Med. 43, 1441–1452. 10.1007/s00134-017-4928-4 28917004

[B66] Dridi H. Yehya M. Barsotti R. Liu Y. Reiken S. Azria L. (2023). Aberrant mitochondrial dynamics contributes to diaphragmatic weakness induced by mechanical ventilation. PNAS Nexus 2, pgad336. 10.1093/pnasnexus/pgad336 PMC10635656 37954156

[B67] Du H. Ma Y. Wang X. Zhang Y. Zhu L. Shi S. (2023). Advanced glycation end products induce skeletal muscle atrophy and insulin resistance via activating ROS-mediated ER stress PERK/FoxO1 signaling. Am. J. Physiol. Endocrinol. Metab. 324, E279–E287. 10.1152/ajpendo.00218.2022 36724125

[B68] Dumont N. A. Bentzinger C. F. Sincennes M. C. Rudnicki M. A. (2015a). Satellite cells and skeletal muscle regeneration. Compr. Physiol. 5, 1027–1059. 10.1002/cphy.c140068 26140708

[B69] Dumont N. A. Wang Y. X. von Maltzahn J. Pasut A. Bentzinger C. F. Brun C. E. (2015b). Dystrophin expression in muscle stem cells regulates their polarity and asymmetric division. Nat. Med. 21, 1455–1463. 10.1038/nm.3990 26569381 PMC4839960

[B70] Egger M. Finsterhölzl M. Farabegoli D. Wippenbeck F. Schlutt M. Müller F. (2024). Comprehensive assessment and progression of health status during neurorehabilitation in survivors of critical illness: a prospective cohort study. Ann. Intensive Care 14, 175. 10.1186/s13613-024-01396-x PMC11599680 39589665

[B71] Egger M. Finsterhölzl M. Wippenbeck F. Müller F. Jahn K. Huge V. (2026). Activities of daily living, physical activity, and care situation in chronic critical illness survivors one year after disease onset: a prospective cohort study. J. Crit. Care 92, 155396. 10.1016/j.jcrc.2025.155396 41354010

[B72] Eliazer S. Muncie J. M. Christensen J. Sun X. D'Urso R. S. Weaver V. M. (2019). Wnt4 from the niche controls the mechano-properties and quiescent state of muscle stem cells. Cell. Stem Cell. 25, 654–665.e4. 10.1016/j.stem.2019.08.007 31495781 PMC6842437

[B73] Eliazer S. Sun X. Barruet E. Brack A. S. (2022). Heterogeneous levels of delta-like 4 within a multinucleated niche cell maintains muscle stem cell diversity. eLife 11, e68180. 10.7554/eLife.68180 PMC9803355 36583937

[B74] Elkalawy H. Sekhar P. Abosena W. (2023). Early detection and assessment of intensive care unit-acquired weakness: a comprehensive review. Acute Crit. Care 38, 409–424. 10.4266/acc.2023.00703 PMC10718497 38052508

[B75] Espino-Gonzalez E. Dalbram E. Mounier R. Gondin J. Farup J. Jessen N. (2024). Impaired skeletal muscle regeneration in diabetes: from cellular and molecular mechanisms to novel treatments. Cell. Metab. 36, 1204–1236. 10.1016/j.cmet.2024.02.014 38490209

[B76] Fan E. Cheek F. Chlan L. Gosselink R. Hart N. Herridge M. S. (2014). An official American thoracic society clinical practice guideline: the diagnosis of intensive care unit-acquired weakness in adults. Am. J. Respir. Crit. Care Med. 190, 1437–1446. 10.1164/rccm.201411-2011ST 25496103

[B77] Feige P. Brun C. E. Ritso M. Rudnicki M. A. (2018). Orienting muscle stem cells for regeneration in homeostasis, aging, and disease. Cell. Stem Cell. 23, 653–664. 10.1016/j.stem.2018.10.006 30388423 PMC6262894

[B78] Forcales S. V. Albini S. Giordani L. Malecova B. Cignolo L. Chernov A. (2012). Signal-dependent incorporation of MyoD-BAF60C into BRG1-based SWI/SNF chromatin-remodelling complex. EMBO J. 31, 301–316. 10.1038/emboj.2011.391 22068056 PMC3261556

[B79] Friedrich O. Reid M. B. Van den Berghe G. Vanhorebeek I. Hermans G. Rich M. M. (2015). The sick and the weak: neuropathies/myopathies in the critically ill. Physiol. Rev. 95, 1025–1109. 10.1152/physrev.00028.2014 PMC4491544 26133937

[B80] Fuentes-Aspe R. Gutierrez-Arias R. González-Seguel F. Marzuca-Nassr G. N. Torres-Castro R. Najum-Flores J. (2024). Which factors are associated with acquired weakness in the ICU? An overview of systematic reviews and meta-analyses. J. Intensive Care 12, 33. 10.1186/s40560-024-00744-0 PMC11375885 39232808

[B81] Furuichi Y. Kawabata Y. Aoki M. Mita Y. Fujii N. L. Manabe Y. (2021). Excess glucose impedes the proliferation of skeletal muscle satellite cells under adherent culture conditions. Front. Cell. Dev. Biol. 9, 640399. 10.3389/fcell.2021.640399 PMC7957019 33732705

[B82] García-Prat L. Martínez-Vicente M. Perdiguero E. Ortet L. Rodríguez-Ubreva J. Rebollo E. (2016). Autophagy maintains stemness by preventing senescence. Nature 529, 37–42. 10.1038/nature16187 26738589

[B83] Genserová L. Duška F. Krajčová A. (2024). β-Hydroxybutyrate exposure restores mitochondrial function in skeletal muscle satellite cells of critically ill patients. Clin. Nutr. 43, 1250–1260. 10.1016/j.clnu.2024.04.009 38653008

[B84] Gentile L. F. Cuenca A. G. Efron P. A. Ang D. Bihorac A. McKinley B. A. (2012). Persistent inflammation and immunosuppression: a common syndrome and new horizon for surgical intensive care. J. Trauma Acute Care Surg. 72, 1491–1501. 10.1097/TA.0b013e318256e000 22695412 PMC3705923

[B85] Gillies A. R. Lieber R. L. (2011). Structure and function of the skeletal muscle extracellular matrix. Muscle Nerve 44, 318–331. 10.1002/mus.22094 21949456 PMC3177172

[B86] Goldenberg N. M. Steinberg B. E. Slutsky A. S. Lee W. L. (2011). Broken barriers: a new take on sepsis pathogenesis. Sci. Transl. Med. 3, 88ps25. 10.1126/scitranslmed.3002011 21697528

[B87] Gopinath S. D. Webb A. E. Brunet A. Rando T. A. (2014). FoxO3 promotes quiescence in adult muscle stem cells during the process of self-renewal. Stem Cell. Rep. 2, 414–426. 10.1016/j.stemcr.2014.02.002 24749067 PMC3986584

[B88] Han L. Wang G. Zhou S. Situ C. He Z. Li Y. (2022). Muscle satellite cells are impaired in type 2 diabetic mice by elevated extracellular adenosine. Cell. Rep. 39, 110884. 10.1016/j.celrep.2022.110884 35649375

[B89] He W. A. Berardi E. Cardillo V. M. Acharyya S. Aulino P. Thomas-Ahner J. (2013). NF-κB-mediated Pax7 dysregulation in the muscle microenvironment promotes cancer cachexia. J. Clin. Invest. 123, 4821–4835. 10.1172/jci68523 24084740 PMC3809785

[B90] Hendrickse P. Degens H. (2019). The role of the microcirculation in muscle function and plasticity. J. Muscle Res. Cell. Motil. 40, 127–140. 10.1007/s10974-019-09520-2 31165949 PMC6726668

[B91] Hendrickse P. W. Wüst R. C. I. Ganse B. Giakoumaki I. Rittweger J. Bosutti A. (2022). Capillary rarefaction during bed rest is proportionally less than fibre atrophy and loss of oxidative capacity. J. Cachexia Sarcopenia Muscle 13, 2712–2723. 10.1002/jcsm.13072 PMC9745458 36102002

[B92] Henze H. Hüttner S. S. Koch P. Schüler S. C. Groth M. von Eyss B. (2024). Denervation alters the secretome of myofibers and thereby affects muscle stem cell lineage progression and functionality. NPJ Regen. Med. 9, 10. 10.1038/s41536-024-00353-3 PMC10904387 38424446

[B93] Hermans G. Van den Berghe G. (2015). Clinical review: intensive care unit acquired weakness. Crit. Care 19, 274. 10.1186/s13054-015-0993-7 26242743 PMC4526175

[B94] Hermans G. De Jonghe B. Bruyninckx F. Van den Berghe G. (2008). Clinical review: critical illness polyneuropathy and myopathy. Crit. Care 12, 238. 10.1186/cc7100 19040777 PMC2646339

[B95] Hermans G. Casaer M. P. Clerckx B. Güiza F. Vanhullebusch T. Derde S. (2013). Effect of tolerating macronutrient deficit on the development of intensive-care unit acquired weakness: a subanalysis of the EPaNIC trial. Lancet Respir. Med. 1, 621–629. 10.1016/S2213-2600(13)70183-8 24461665

[B96] Hermans G. Van Mechelen H. Clerckx B. Vanhullebusch T. Mesotten D. Wilmer A. (2014). Acute outcomes and 1-year mortality of intensive care unit-acquired weakness: a cohort study and propensity-matched analysis. Am. J. Respir. Crit. Care Med. 190, 410–420. 10.1164/rccm.201312-2257OC 24825371

[B97] Herridge M. S. Tansey C. M. Matté A. Tomlinson G. Diaz-Granados N. Cooper A. (2011). Functional disability 5 years after acute respiratory distress syndrome. N. Engl. J. Med. 364, 1293–1304. 10.1056/NEJMoa1011802 21470008

[B98] Heyland D. K. Patel J. Compher C. Rice T. W. Bear D. E. Lee Z. Y. (2023). The effect of higher protein dosing in critically ill patients with high nutritional risk (EFFORT protein): an international, multicentre, pragmatic, registry-based randomised trial. Lancet 401, 568–576. 10.1016/S0140-6736(22)02469-2 36708732

[B99] Ho T. L. Lai Y. L. Hsu C. J. Su C. M. Tang C. H. (2023). High-mobility group box-1 impedes skeletal muscle regeneration via downregulation of Pax-7 synthesis by increasing miR-342-5p expression. Aging (Albany NY) 15, 12618–12632. 10.18632/aging.205202 PMC10683625 37963838

[B100] Hodgson C. L. Bailey M. Bellomo R. Brickell K. Broadley T. Buhr H. (2022). Early active mobilization during mechanical ventilation in the ICU. N. Engl. J. Med. 387, 1747–1758. 10.1056/NEJMoa2209083 36286256

[B101] Hooijman P. E. Beishuizen A. Witt C. C. de Waard M. C. Girbes A. R. Spoelstra-de Man A. M. (2015). Diaphragm muscle fiber weakness and ubiquitin-proteasome activation in critically ill patients. Am. J. Respir. Crit. Care Med. 191, 1126–1138. 10.1164/rccm.201412-2214OC 25760684 PMC4451621

[B102] Hyatt H. W. Ozdemir M. Yoshihara T. Nguyen B. L. Deminice R. Powers S. K. (2021). Calpains play an essential role in mechanical ventilation-induced diaphragmatic weakness and mitochondrial dysfunction. Redox Biol. 38, 101802. 10.1016/j.redox.2020.101802 33279868 PMC7724197

[B103] Inoue S. Nakanishi N. Amaya F. Fujinami Y. Hatakeyama J. Hifumi T. (2024). Post-intensive care syndrome: recent advances and future directions. Acute Med. Surg. 11, e929. 10.1002/ams2.929 PMC10879727 38385144

[B104] Intiso D. Centra A. M. Bartolo M. Gatta M. T. Gravina M. Di Rienzo F. (2022). Recovery and long term functional outcome in people with critical illness polyneuropathy and myopathy: a scoping review. BMC Neurol. 22, 50. 10.1186/s12883-022-02570-z PMC8831873 35148710

[B105] Jaber S. Petrof B. J. Jung B. Chanques G. Berthet J. P. Rabuel C. (2011). Rapidly progressive diaphragmatic weakness and injury during mechanical ventilation in humans. Am. J. Respir. Crit. Care Med. 183, 364–371. 10.1164/rccm.201004-0670OC 20813887

[B106] Joffre J. Hellman J. Ince C. Ait-Oufella H. (2020). Endothelial responses in sepsis. Am. J. Respir. Crit. Care Med. 202, 361–370. 10.1164/rccm.201910-1911TR 32101446

[B107] Johnson A. L. Kamal M. Parise G. (2023). The role of supporting cell populations in satellite cell mediated muscle repair. Cells 12, 1968. 10.3390/cells12151968 PMC10417507 37566047

[B108] Jones T. K. Reilly J. P. Anderson B. J. Miano T. A. Dunn T. G. Weisman A. R. (2022). Elevated plasma levels of matrix metalloproteinase-3 and tissue-inhibitor of matrix metalloproteinases-1 associate with organ dysfunction and mortality in sepsis. Shock 57, 41–47. 10.1097/SHK.0000000000001833 34265829 PMC8663538

[B109] Kingren M. S. Keeble A. R. Galvan-Lara A. M. Ogle J. M. Ungvári Z. St Clair D. K. (2024). Post-sepsis chronic muscle weakness can be prevented by pharmacological protection of mitochondria. Mol. Med. 30, 221. 10.1186/s10020-024-00982-w PMC11577827 39563237

[B110] Klawitter F. Ehler J. Bajorat R. Patejdl R. (2023). Mitochondrial dysfunction in intensive care unit-acquired weakness and critical illness myopathy: a narrative review. Int. J. Mol. Sci. 24, 5516. 10.3390/ijms24065516 PMC10052131 36982590

[B111] Klawitter F. Laukien F. Fischer D. C. Rahn A. Porath K. Danckert L. (2025). Longitudinal assessment of blood-based inflammatory, neuromuscular, and neurovascular biomarker profiles in intensive care unit-acquired weakness: a prospective single-center cohort study. Neurocrit. Care 42, 118–130. 10.1007/s12028-024-02050-x PMC11811256 38982001

[B112] Koike H. Sugimura M. Ouchi R. Yoshimoto Y. Manabe I. Oishi Y. (2025). Macrophage subpopulation promotes skeletal muscle regeneration through HGF/Met signaling-mediated skeletal muscle stem cell proliferation. Aging Cell. 24, e70042. 10.1111/acel.70042 40132988 PMC12151914

[B113] Komaki M. Asakura A. Rudnicki M. A. Sodek J. Cheifetz S. (2004). MyoD enhances BMP7-induced osteogenic differentiation of myogenic cell cultures. J. Cell. Sci. 117, 1457–1468. 10.1242/jcs.00965 15020674

[B114] Kostallari E. Baba-Amer Y. Alonso-Martin S. Ngoh P. Relaix F. Lafuste P. (2015). Pericytes in the myovascular niche promote post-natal myofiber growth and satellite cell quiescence. Development 142, 1242–1253. 10.1242/dev.115386 25742797

[B115] Krajčová A. Němcová V. Škrha P. Genserová L. Musilová A. Fric M. (2026). Critical illness impairs coordinated myogenesis: a prospective longitudinal study of skeletal muscle progenitor cell biology using serial muscle biopsies (SATELLITE study). Crit. Care 30, 443. 10.1186/s13054-026-06131-5 42304498 PMC13501688

[B116] Kuang S. Kuroda K. Le Grand F. Rudnicki M. A. (2007). Asymmetric self-renewal and commitment of satellite stem cells in muscle. Cell. 129, 999–1010. 10.1016/j.cell.2007.03.044 17540178 PMC2718740

[B117] Kuswanto W. Burzyn D. Panduro M. Wang K. K. Jang Y. C. Wagers A. J. (2016). Poor repair of skeletal muscle in aging mice reflects a defect in local, interleukin-33-dependent accumulation of regulatory t cells. Immunity 44, 355–367. 10.1016/j.immuni.2016.01.009 26872699 PMC4764071

[B118] Lad H. Saumur T. M. Herridge M. S. Dos Santos C. C. Mathur S. Batt J. (2020). Intensive care unit-acquired weakness: not just another muscle atrophying condition. Int. J. Mol. Sci. 21, 7840. 10.3390/ijms21217840 33105809 PMC7660068

[B119] Langley B. Thomas M. Bishop A. Sharma M. Gilmour S. Kambadur R. (2002). Myostatin inhibits myoblast differentiation by down-regulating MyoD expression. J. Biol. Chem. 277, 49831–49840. 10.1074/jbc.M204291200 12244043

[B120] Larouche J. A. Mohiuddin M. Choi J. J. Ulintz P. J. Fraczek P. Sabin K. (2021). Murine muscle stem cell response to perturbations of the neuromuscular junction are attenuated with aging. eLife 10, e66749. 10.7554/eLife.66749 34323217 PMC8360658

[B121] Latroche C. Weiss-Gayet M. Muller L. Gitiaux C. Leblanc P. Liot S. (2017). Coupling between myogenesis and angiogenesis during skeletal muscle regeneration is stimulated by restorative macrophages. Stem Cell. Rep. 9, 2018–2033. 10.1016/j.stemcr.2017.10.027 PMC5785732 29198825

[B122] Latronico N. Rasulo F. A. Eikermann M. Piva S. (2023). Illness weakness, polyneuropathy and myopathy: diagnosis, treatment, and long-term outcomes. Crit. Care 27, 439. 10.1186/s13054-023-04676-3 37957759 PMC10644573

[B123] Le Grand F. Jones A. E. Seale V. Scimè A. Rudnicki M. A. (2009). Wnt7a activates the planar cell polarity pathway to drive the symmetric expansion of satellite stem cells. Cell. Stem Cell. 4, 535–547. 10.1016/j.stem.2009.03.013 19497282 PMC2743383

[B124] Lees M. J. Prado C. M. Wischmeyer P. E. Phillips S. M. (2025). Skeletal muscle: a critical organ for survival and recovery in critical illness. Crit. Care Clin. 41, 299–312. 10.1016/j.ccc.2024.08.011 40021281

[B125] Lemos D. R. Babaeijandaghi F. Low M. Chang C. K. Lee S. T. Fiore D. (2015). Nilotinib reduces muscle fibrosis in chronic muscle injury by promoting TNF-mediated apoptosis of fibro/adipogenic progenitors. Nat. Med. 21, 786–794. 10.1038/nm.3869 26053624

[B126] Levine S. Nguyen T. Taylor N. Friscia M. E. Budak M. T. Rothenberg P. (2008). Rapid disuse atrophy of diaphragm fibers in mechanically ventilated humans. N. Engl. J. Med. 358, 1327–1335. 10.1056/NEJMoa070447 18367735

[B127] Li W. Moylan J. S. Chambers M. A. Smith J. Reid M. B. (2009). Interleukin-1 stimulates catabolism in C2C12 myotubes. Am. J. Physiol. Cell. Physiol. 297, C706–C714. 10.1152/ajpcell.00626.2008 19625606 PMC2740393

[B128] Li L. Liu H. Tao W. Wen S. Fu X. Yu S. (2021). Pharmacological inhibition of HMGB1 prevents muscle wasting. Front. Pharmacol. 12, 731386. 10.3389/fphar.2021.731386 PMC8637759 34867338

[B129] Li Y. Dörmann N. Brinschwitz B. Kny M. Martin E. Bartels K. (2023). SPSB1-mediated inhibition of TGF-β receptor-II impairs myogenesis in inflammation. J. Cachexia Sarcopenia Muscle 14, 1721–1736. 10.1002/jcsm.13252 37209006 PMC10401548

[B130] Li L. Jiang F. Hao W. Wang Y. Li Y. Zhang D. (2024). Single-nucleus transcriptomic profiling of the diaphragm during mechanical ventilation. Sci. Rep. 14, 31181. 10.1038/s41598-024-82530-4 39732791 PMC11682457

[B131] Liu W. Wei-LaPierre L. Klose A. Dirksen R. T. Chakkalakal J. V. (2015). Inducible depletion of adult skeletal muscle stem cells impairs the regeneration of neuromuscular junctions. eLife 4, e09221. 10.7554/eLife.09221 26312504 PMC4579298

[B132] Liu W. Klose A. Forman S. Paris N. D. Wei-LaPierre L. Cortés-Lopéz M. (2017). Loss of adult skeletal muscle stem cells drives age-related neuromuscular junction degeneration. eLife 6, e26464. 10.7554/eLife.26464 28583253 PMC5462534

[B133] Liu S. D'Amico D. Shankland E. Bhayana S. Garcia J. M. Aebischer P. (2022). Effect of urolithin A supplementation on muscle endurance and mitochondrial health in older adults: a randomized clinical trial. JAMA Netw. Open 5, e2144279. 10.1001/jamanetworkopen.2021.44279 35050355 PMC8777576

[B134] Londhe P. Davie J. K. (2013). Interferon-γ resets muscle cell fate by stimulating the sequential recruitment of JARID2 and PRC2 to promoters to repress myogenesis. Sci. Signal. 6, ra107. 10.1126/scisignal.2004633 24327761 PMC4000160

[B135] Londhe P. Guttridge D. C. (2015). Inflammation induced loss of skeletal muscle. Bone 80, 131–142. 10.1016/j.bone.2015.03.015 26453502 PMC4600538

[B136] Loreti M. Sacco A. (2022). The jam session between muscle stem cells and the extracellular matrix in the tissue microenvironment. NPJ regen. Med 7, 16. 10.1038/s41536-022-00204-z PMC8854427 35177651

[B137] Lukjanenko L. Jung M. J. Hegde N. Perruisseau-Carrier C. Migliavacca E. Rozo M. (2016). Loss of fibronectin from the aged stem cell niche affects the regenerative capacity of skeletal muscle in mice. Nat. Med. 22, 897–905. 10.1038/nm.4126 27376579 PMC5467443

[B138] Ma L. Meng Y. An Y. Han P. Zhang C. Yue Y. (2024). Single-cell RNA-seq reveals novel interaction between muscle satellite cells and fibro-adipogenic progenitors mediated with FGF7 signalling. J. Cachexia Sarcopenia Muscle 15, 1388–1403. 10.1002/jcsm.13484 38751367 PMC11294021

[B139] Madaro L. Passafaro M. Sala D. Etxaniz U. Lugarini F. Proietti D. (2018). Denervation-activated STAT3-IL-6 signalling in fibro-adipogenic progenitors promotes myofibres atrophy and fibrosis. Nat. Cell. Biol. 20, 917–927. 10.1038/s41556-018-0151-y 30050118 PMC6145844

[B140] Mart M. F. Gordon J. I. González-Seguel F. Mayer K. P. Brummel N. (2026). Muscle dysfunction and physical recovery after critical illness. J. Intensive Care Med. 41, 214–230. 10.1177/08850666251317467 39905778 PMC12852493

[B141] Martin G. Asensi V. Montes A. H. Collazos J. Alvarez V. Carton J. A. (2014). Role of plasma matrix-metalloproteases (MMPs) and their polymorphisms (SNPs) in sepsis development and outcome in ICU patients. Sci. Rep. 4, 5002. 10.1038/srep05002 24833564 PMC4023133

[B142] Mathur S. Maia N. P. S. De Paula Ferreira M. Martin C. Doherty C. Correa J. (2025). A novel short-course, low-intensity blood-flow-restricted exercise (BFRE) regimen to study satellite cell function in critical illness survivors with sustained muscle atrophy following intensive care unit-acquired weakness (ICUAW). Front. Physiol. 16, 1553471. 10.3389/fphys.2025.1553471 PMC12214465 40606234

[B143] Mayer K. P. Ismaeel A. Kalema A. G. Montgomery-Yates A. A. Soper M. K. Kern P. A. (2024). Persistent fatigue, weakness, and aberrant muscle mitochondria in survivors of critical COVID-19. Crit. Care Explor 6, e1164. 10.1097/CCE.0000000000001164 39412208 PMC11487221

[B144] McCroskery S. Thomas M. Maxwell L. Sharma M. Kambadur R. (2003). Myostatin negatively regulates satellite cell activation and self-renewal. J. Cell. Biol. 162, 1135–1147. 10.1083/jcb.200207056 12963705 PMC2172861

[B145] McKellar D. W. Walter L. D. Song L. T. Mantri M. Wang M. F. Z. De Vlaminck I. (2021). Large-scale integration of single-cell transcriptomic data captures transitional progenitor states in mouse skeletal muscle regeneration. Commun. Biol 4, 1280. 10.1038/s42003-021-02810-x PMC8589952 34773081

[B146] McKenna C. F. Fry C. S. (2017). Altered satellite cell dynamics accompany skeletal muscle atrophy during chronic illness, disuse, and aging. Curr. Opin. Clin. Nutr. Metab. Care 20, 447–452. 10.1097/MCO.0000000000000409 28795971 PMC5810415

[B147] Mendelson A. A. Erickson D. Villar R. (2023). The role of the microcirculation and integrative cardiovascular physiology in the pathogenesis of ICU-acquired weakness. Front. Physiol. 14, 1170429. 10.3389/fphys.2023.1170429 37234410 PMC10206327

[B148] Milan G. Romanello V. Pescatore F. Armani A. Paik J. H. Frasson L. (2015). Regulation of autophagy and the ubiquitin-proteasome system by the FoxO transcriptional network during muscle atrophy. Nat. Commun. 6, 6670. 10.1038/ncomms7670 25858807 PMC4403316

[B149] Mira J. C. Brakenridge S. C. Moldawer L. L. Moore F. A. (2017a). Persistent inflammation, immunosuppression and catabolism syndrome. Crit. Care Clin. 33, 245–258. 10.1016/j.ccc.2016.12.001 28284293 PMC5351769

[B150] Mira J. C. Gentile L. F. Mathias B. J. Efron P. A. Brakenridge S. C. Mohr A. M. (2017b). Sepsis pathophysiology, chronic critical illness, and persistent inflammation-immunosuppression and catabolism syndrome. Crit. Care Med. 45, 253–262. 10.1097/CCM.0000000000002074 27632674 PMC5243156

[B151] Moss M. Nordon-Craft A. Malone D. Van Pelt D. Frankel S. K. Warner M. L. (2016). A randomized trial of an intensive physical therapy program for patients with acute respiratory failure. Am. J. Respir. Crit. Care Med. 193, 1101–1110. 10.1164/rccm.201505-1039OC 26651376 PMC4872662

[B152] Motohashi N. Asakura A. (2014). Muscle satellite cell heterogeneity and self-renewal. Front. Cell. Dev. Biol. 2, 1. 10.3389/fcell.2014.00001 25364710 PMC4206996

[B153] Mourikis P. Sambasivan R. Castel D. Rocheteau P. Bizzarro V. Tajbakhsh S. (2012). A critical requirement for notch signaling in maintenance of the quiescent skeletal muscle stem cell state. Stem Cells 30, 243–252. 10.1002/stem.775 22069237

[B154] Muñoz-Cánoves P. Scheele C. Pedersen B. K. Serrano A. L. (2013). Interleukin-6 myokine signaling in skeletal muscle: a double-edged sword? FEBS J. 280, 4131–4148. 10.1111/febs.12338 23663276 PMC4163639

[B155] Munroe M. Dvoretskiy S. Lopez A. Leong J. Dyle M. C. Kong H. (2019). Pericyte transplantation improves skeletal muscle recovery following hindlimb immobilization. FASEB J. 33, 7694–7706. 10.1096/fj.201802580r 31021652 PMC6529341

[B156] Murray I. R. Baily J. E. Chen W. C. W. Dar A. Gonzalez Z. N. Jensen A. R. (2017). Skeletal and cardiac muscle pericytes: functions and therapeutic potential. Pharmacol. Ther. 171, 65–74. 10.1016/j.pharmthera.2016.09.005 27595928 PMC5726267

[B157] Nederveen J. P. Joanisse S. Snijders T. Ivankovic V. Baker S. K. Phillips S. M. (2016). Skeletal muscle satellite cells are located at a closer proximity to capillaries in healthy young compared with older men. J. Cachexia Sarcopenia Muscle 7, 547–554. 10.1002/jcsm.12105 27239425 PMC4864218

[B158] Needham D. M. Davidson J. Cohen H. Hopkins R. O. Weinert C. Wunsch H. (2012). Improving long-term outcomes after discharge from intensive care unit: report from a stakeholders' conference. Crit. Care Med. 40, 502–509. 10.1097/CCM.0b013e318232da75 21946660

[B159] Okafor A. E. Lin X. Situ C. Wei X. Xiang Y. Wei X. (2023). Single-cell chromatin accessibility profiling reveals a self-renewing muscle satellite cell state. J. Cell. Biol. 222, e202211073. 10.1083/jcb.202211073 37382627 PMC10309185

[B160] Ono Y. Sakamoto K. (2017). Lipopolysaccharide inhibits myogenic differentiation of C2C12 myoblasts through the toll-like receptor 4-nuclear factor-κB signaling pathway and myoblast-derived tumor necrosis factor-α. PLoS One 12, e0182040. 10.1371/journal.pone.0182040 28742154 PMC5524356

[B161] Osiaevi I. Schulze A. Evers G. Harmening K. Vink H. Kümpers P. (2023). Persistent capillary rarefication in long COVID syndrome. Angiogenesis 26, 53–61. 10.1007/s10456-022-09850-9 35951203 PMC9366128

[B162] Owen A. M. Patel S. P. Smith J. D. Balasuriya B. K. Mori S. F. Hawk G. S. (2019). Chronic muscle weakness and mitochondrial dysfunction in the absence of sustained atrophy in a preclinical sepsis model. eLife 8, e49920. 10.7554/eLife.49920 31793435 PMC6890461

[B163] Palacios D. Mozzetta C. Consalvi S. Caretti G. Saccone V. Proserpio V. (2010). TNF/p38α/Polycomb signaling to Pax7 locus in satellite cells links inflammation to the epigenetic control of muscle regeneration. Cell. Stem Cell. 7, 455–469. 10.1016/j.stem.2010.08.013 20887952 PMC2951277

[B164] Peñuelas O. Keough E. López-Rodríguez L. Carriedo D. Gonçalves G. Barreiro E. (2019). Ventilator-induced diaphragm dysfunction: translational mechanisms lead to therapeutical alternatives in the critically ill. Intensive Care Med. Exp. 7, 48. 10.1186/s40635-019-0259-9 31346802 PMC6658639

[B165] Pierre A. Favory R. Bourel C. Howsam M. Romien R. Lancel S. (2025a). Muscle weakness after critical illness: unravelling biological mechanisms and clinical hurdles. Crit. Care 29, 248. 10.1186/s13054-025-05462-z PMC12175454 40528196

[B166] Pierre A. Favory R. Brassart B. Bourel C. Romien R. Normandin S. (2025b). Sepsis induces long-term muscle and mitochondrial dysfunction due to autophagy disruption amenable by urolithin A. J. Cachexia Sarcopenia Muscle 16, e70041. 10.1002/jcsm.70041 PMC12356704 40817441

[B167] Polkey M. I. Praestgaard J. Berwick A. Franssen F. M. E. Singh D. Steiner M. C. (2019). Activin type II receptor blockade for treatment of muscle depletion in chronic obstructive pulmonary disease. A randomized trial. Am. J. Respir. Crit. Care Med. 199, 313–320. 10.1164/rccm.201802-0286OC 30095981 PMC6363975

[B168] Powers S. K. Wiggs M. P. Sollanek K. J. Smuder A. J. (2013). Ventilator-induced diaphragm dysfunction: cause and effect. Am. J. Physiol. Regul. Integr. Comp. Physiol. 305, R464–R477. 10.1152/ajpregu.00231.2013 23842681

[B169] Price F. D. von Maltzahn J. Bentzinger C. F. Dumont N. A. Yin H. Chang N. C. (2014). Inhibition of JAK-STAT signaling stimulates adult satellite cell function. Nat. Med. 20, 1174–1181. 10.1038/nm.3655 25194569 PMC4191983

[B170] Puthucheary Z. A. Rawal J. McPhail M. Connolly B. Ratnayake G. Chan P. (2013). Acute skeletal muscle wasting in critical illness. JAMA 310, 1591–1600. 10.1001/jama.2013.278481 24108501

[B171] Ratnayake D. Nguyen P. D. Rossello F. J. Wimmer V. C. Tan J. L. Galvis L. A. (2021). Macrophages provide a transient muscle stem cell niche via NAMPT secretion. Nature 591, 281–287. 10.1038/s41586-021-03199-7 33568815

[B172] Rayagiri S. S. Ranaldi D. Raven A. Mohamad Azhar N. I. F. Lefebvre O. Zammit P. S. (2018). Basal lamina remodeling at the skeletal muscle stem cell niche mediates stem cell self-renewal. Nat. Commun. 9, 1075. 10.1038/s41467-018-03425-3 29540680 PMC5852002

[B173] Relaix F. Zammit P. S. (2012). Satellite cells are essential for skeletal muscle regeneration: the cell on the edge returns centre stage. Development 139, 2845–2856. 10.1242/dev.069088 22833472

[B174] Rocheteau P. Chatre L. Briand D. Mebarki M. Jouvion G. Bardon J. (2015). Sepsis induces long-term metabolic and mitochondrial muscle stem cell dysfunction amenable by mesenchymal stem cell therapy. Nat. Commun. 6, 10145. 10.1038/ncomms10145 26666572 PMC4682118

[B175] Rodriguez B. Schefold J. C. Z'Graggen W. J. (2024). Diagnosis of “intensive care unit-acquired weakness” and “critical illness myopathy”: do the diagnostic criteria need to be revised? Clin. Neurophysiol. Pract. 9, 236–241. 10.1016/j.cnp.2024.08.002 39282048 PMC11402439

[B176] Rooks D. Swan T. Goswami B. Filosa L. A. Bunte O. Panchaud N. (2020). Bimagrumab vs optimized standard of care for treatment of sarcopenia in community-dwelling older adults: a randomized clinical trial. JAMA Netw. Open 3, e2020836. 10.1001/jamanetworkopen.2020.20836 33074327 PMC7573681

[B177] Ryall J. G. Dell'Orso S. Derfoul A. Juan A. Zare H. Feng X. (2015). The NAD(+)-Dependent SIRT1 deacetylase translates a metabolic switch into regulatory epigenetics in skeletal muscle stem cells. Cell. Stem Cell. 16, 171–183. 10.1016/j.stem.2014.12.004 25600643 PMC4320668

[B178] Saccheri C. Morawiec E. Delemazure J. Mayaux J. Dubé B. P. Similowski T. (2020). ICU-acquired weakness, diaphragm dysfunction and long-term outcomes of critically ill patients. Ann. Intensive Care 10, 1. 10.1186/s13613-019-0618-4 31900667 PMC6942110

[B179] Sack K. D. Kellum J. A. Parikh S. M. (2020). The angiopoietin-Tie2 pathway in critical illness. Crit. Care Clin. 36, 201–216. 10.1016/j.ccc.2019.12.003 32172809 PMC8843037

[B180] Sandri M. Sandri C. Gilbert A. Skurk C. Calabria E. Picard A. (2004). FoxO transcription factors induce the atrophy-related ubiquitin ligase atrogin-1 and cause skeletal muscle atrophy. Cell. 117, 399–412. 10.1016/S0092-8674(04)00400-3 15109499 PMC3619734

[B181] Schefold J. C. Wollersheim T. Grunow J. J. Luedi M. M. Z'Graggen W. J. Weber-Carstens S. (2020). Muscular weakness and muscle wasting in the critically ill. J. Cachexia Sarcopenia Muscle 11, 1399–1412. 10.1002/jcsm.12620 32893974 PMC7749542

[B182] Scheibenbogen C. Wirth K. J. (2025). Key pathophysiological role of skeletal muscle disturbance in post COVID and myalgic encephalomyelitis/chronic fatigue syndrome (me/cfs): accumulated evidence. J. Cachexia Sarcopenia Muscle 16, e13669. 10.1002/jcsm.13669 PMC11671797 39727052

[B183] Schmitt R. E. Dasgupta A. Arneson-Wissink P. C. Datta S. Ducharme A. M. Doles J. D. (2023). Muscle stem cells contribute to long-term tissue repletion following surgical sepsis. J. Cachexia Sarcopenia Muscle 14, 1424–1440. 10.1002/jcsm.13214 36883680 PMC10235871

[B184] Serrano A. L. Baeza-Raja B. Perdiguero E. Jardí M. Muñoz-Cánoves P. (2008). Interleukin-6 is an essential regulator of satellite cell-mediated skeletal muscle hypertrophy. Cell. Metab. 7, 33–44. 10.1016/j.cmet.2007.11.011 18177723

[B185] Shi Z. van den Berg M. Bogaards S. Conijn S. Paul M. Beishuizen A. (2023). Replacement fibrosis in the diaphragm of mechanically ventilated critically ill patients. Am. J. Respir. Crit. Care Med. 207, 351–354. 10.1164/rccm.202208-1608LE 36178289 PMC12042655

[B186] Silver J. S. Günay K. A. Cutler A. A. Vogler T. O. Brown T. E. Pawlikowski B. T. (2021). Injury-mediated stiffening persistently activates muscle stem cells through YAP and TAZ mechanotransduction. Sci. Adv. 7, eabe4501. 10.1126/sciadv.abe4501 33712460 PMC7954458

[B187] Simone C. Forcales S. V. Hill D. A. Imbalzano A. N. Latella L. Puri P. L. (2004). p38 pathway targets SWI-SNF chromatin-remodeling complex to muscle-specific loci. Nat. Genet. 36, 738–743. 10.1038/ng1378 15208625

[B188] Singh A. D'Amico D. Andreux P. A. Fouassier A. M. Blanco-Bose W. Evans M. (2022). Urolithin A improves muscle strength, exercise performance, and biomarkers of mitochondrial health in a randomized trial in middle-aged adults. Cell. Rep. Med. 3, 100633. 10.1016/j.xcrm.2022.100633 35584623 PMC9133463

[B189] Smuder A. J. Sollanek K. J. Nelson W. B. Min K. Talbert E. E. Kavazis A. N. (2018). Crosstalk between autophagy and oxidative stress regulates proteolysis in the diaphragm during mechanical ventilation. Free Radic. Biol. Med. 115, 179–190. 10.1016/j.freeradbiomed.2017.11.025 29197632 PMC5767544

[B190] Snijders T. Wall B. T. Dirks M. L. Senden J. M. Hartgens F. Dolmans J. (2014). Muscle disuse atrophy is not accompanied by changes in skeletal muscle satellite cell content. Clin. Sci. (Lond) 126, 557–566. 10.1042/cs20130295 24215591

[B191] Snijders T. Nederveen J. P. Joanisse S. Leenders M. Verdijk L. B. van Loon L. J. (2017). Muscle fibre capillarization is a critical factor in muscle fibre hypertrophy during resistance exercise training in older men. J. Cachexia Sarcopenia Muscle 8, 267–276. 10.1002/jcsm.12137 27897408 PMC5377411

[B192] Sobolewska J. Dzialach L. Kuca P. Witek P. (2024). Critical illness-related corticosteroid insufficiency (CIRCI) – an overview of pathogenesis, clinical presentation and management. Front. Endocrinol. (Lausanne) 15, 1473151. 10.3389/fendo.2024.1473151 39574948 PMC11580036

[B193] Song J. Deng T. Yu Q. Luo X. Miao Y. Xie L. (2025). Biomarkers for intensive care unit-acquired weakness: a systematic review for prediction, diagnosis and prognosis. Ann. Intensive Care 15, 86. 10.1186/s13613-025-01500-9 40601169 PMC12222590

[B194] Sousa-Victor P. Gutarra S. García-Prat L. Rodriguez-Ubreva J. Ortet L. Ruiz-Bonilla V. (2014). Geriatric muscle stem cells switch reversible quiescence into senescence. Nature 506, 316–321. 10.1038/nature13013 24522534

[B195] Stearns-Reider K. M. D'Amore A. Beezhold K. Rothrauff B. Cavalli L. Wagner W. R. (2017). Aging of the skeletal muscle extracellular matrix drives a stem cell fibrogenic conversion. Aging Cell. 16, 518–528. 10.1111/acel.12578 28371268 PMC5418187

[B196] Stevens R. D. Marshall S. A. Cornblath D. R. Hoke A. Needham D. M. de Jonghe B. (2009). A framework for diagnosing and classifying intensive care unit-acquired weakness. Crit. Care Med. 37, S299–S308. 10.1097/CCM.0b013e3181b6ef67 20046114

[B197] Straughn A. R. Hindi S. M. Xiong G. Kumar A. (2019). Canonical NF-κB signaling regulates satellite stem cell homeostasis and function during regenerative myogenesis. J. Mol. Cell. Biol. 11, 53–66. 10.1093/jmcb/mjy053 30239789

[B198] Stuelsatz P. Keire P. Almuly R. Yablonka-Reuveni Z. (2012). A contemporary atlas of the mouse diaphragm: myogenicity, vascularity, and the Pax3 connection. J. Histochem. Cytochem. 60, 638–657. 10.1369/0022155412452417 22723526 PMC3524553

[B199] Suetta C. Frandsen U. Mackey A. L. Jensen L. Hvid L. G. Bayer M. L. (2013). Ageing is associated with diminished muscle re-growth and myogenic precursor cell expansion early after immobility-induced atrophy in human skeletal muscle. J. Physiol. 591, 3789–3804. 10.1113/jphysiol.2013.257121 23732643 PMC3752458

[B200] Swartz M. A. Fleury M. E. (2007). Interstitial flow and its effects in soft tissues. Annu. Rev. Biomed. Eng. 9, 229–256. 10.1146/annurev.bioeng.9.060906.151850 17459001

[B201] Taketa T. Uchiyama Y. Miyagi Y. Yamakawa S. Seo T. Yanagida A. (2024). Long-term health-related quality of life and physical function of COVID-19 survivors with ICU-acquired weakness. Prog. Rehabil. Med. 9, 20240012. 10.2490/prm.20240012 38572470 PMC10985412

[B202] Tanaka M. Kim N. C. Draper I. Littel H. R. Gunasekaran M. Turner J. (2026). Deficiency of muscular dystrophy-related gene JAG2 causes NOTCH signaling dysfunction in muscle stem cells. J. Clin. Invest. 136, e198639. 10.1172/JCI198639 PMC13318124 42154534

[B203] Tang A. H. Rando T. A. (2014). Induction of autophagy supports the bioenergetic demands of quiescent muscle stem cell activation. EMBO J. 33, 2782–2797. 10.15252/embj.201488278 25316028 PMC4282556

[B204] Téblick A. Peeters B. Langouche L. Van den Berghe G. (2019). Adrenal function and dysfunction in critically ill patients. Nat. Rev. Endocrinol. 15, 417–427. 10.1038/s41574-019-0185-7 30850749

[B205] Thomas N. T. Confides A. L. Fry C. S. Dupont-Versteegden E. E. (2022). Satellite cell depletion does not affect diaphragm adaptations to hypoxia. J. Appl. Physiol. (1985) 133, 637–646. 10.1152/japplphysiol.00083.2022 35861521 PMC9448290

[B206] Tickle P. G. Hendrickse P. W. Degens H. Egginton S. (2020). Impaired skeletal muscle performance as a consequence of random functional capillary rarefaction can be restored with overload-dependent angiogenesis. J. Physiol. 598, 1187–1203. 10.1113/jp278975 32012275 PMC7154729

[B207] Tidball J. G. (2017). Regulation of muscle growth and regeneration by the immune system. Nat. Rev. Immunol. 17, 165–178. 10.1038/nri.2016.150 28163303 PMC5452982

[B208] Tierney M. T. Aydogdu T. Sala D. Malecova B. Gatto S. Puri P. L. (2014). STAT3 signaling controls satellite cell expansion and skeletal muscle repair. Nat. Med. 20, 1182–1186. 10.1038/nm.3656 25194572 PMC4332844

[B209] Tintignac L. A. Lagirand J. Batonnet S. Sirri V. Leibovitch M. P. Leibovitch S. A. (2005). Degradation of MyoD mediated by the SCF (MAFbx) ubiquitin ligase. J. Biol. Chem. 280, 2847–2856. 10.1074/jbc.M411346200 15531760

[B210] Troy A. Cadwallader A. B. Fedorov Y. Tyner K. Tanaka K. K. Olwin B. B. (2012). Coordination of satellite cell activation and self-renewal by PAR-complex-dependent asymmetric activation of p38α/β MAPK. Cell. Stem Cell. 11, 541–553. 10.1016/j.stem.2012.05.025 23040480 PMC4077199

[B211] Umbrello M. Brogi E. Formenti P. Corradi F. Forfori F. (2024). Ultrasonographic features of muscular weakness and muscle wasting in critically ill patients. J. Clin. Med. 13, 26. 10.3390/jcm13010026 PMC10780243 38202033

[B212] Urciuolo A. Quarta M. Morbidoni V. Gattazzo F. Molon S. Grumati P. (2013). Collagen VI regulates satellite cell self-renewal and muscle regeneration. Nat. Commun. 4, 1964. 10.1038/ncomms2964 23743995 PMC3682802

[B213] Uzun Ayar C. Güiza F. Derese I. Pauwels L. Vander Perre S. Pintelon I. (2025). Altered muscle transcriptome as molecular basis of long-term muscle weakness in survivors from critical illness. Intensive Care Med. 51, 1062–1077. 10.1007/s00134-025-07949-3 40493222 PMC12208968

[B214] Uzun Ayar C. Güiza F. Derese I. Van den Berghe G. Vanhorebeek I. (2026). Association between abnormal DNA methylation and altered transcriptome in muscle five years after critical illness. J. Cachexia Sarcopenia Muscle 17, e70170. 10.1002/jcsm.70170 PMC12813554 41549646

[B215] van den Berg M. Hooijman P. E. Beishuizen A. de Waard M. C. Paul M. A. Hartemink K. J. (2017). Diaphragm atrophy and weakness in the absence of mitochondrial dysfunction in the critically ill. Am. J. Respir. Crit. Care Med. 196, 1544–1558. 10.1164/rccm.201703-0501OC 28787181 PMC5754442

[B216] van den Berghe G. Wouters P. Weekers F. Verwaest C. Bruyninckx F. Schetz M. (2001). Intensive insulin therapy in critically ill patients. N. Engl. J. Med. 345, 1359–1367. 10.1056/NEJMoa011300 11794168

[B217] Vanhorebeek I. Latronico N. Van den Berghe G. (2020). ICU-acquired weakness. Intensive Care Med. 46, 637–653. 10.1007/s00134-020-05944-4 32076765 PMC7224132

[B218] Vanhorebeek I. Casaer M. Gunst J. (2023). Nutrition and autophagy deficiency in critical illness. Curr. Opin. Crit. Care 29, 306–314. 10.1097/MCC.0000000000001056 37306474 PMC10328539

[B219] Verdijk L. B. Koopman R. Schaart G. Meijer K. Savelberg H. H. van Loon L. J. (2007). Satellite cell content is specifically reduced in type II skeletal muscle fibers in the elderly. Am. J. Physiol. Endocrinol. Metab. 292, E151–E157. 10.1152/ajpendo.00278.2006 16926381

[B220] Verma M. Asakura Y. Murakonda B. S. R. Pengo T. Latroche C. Chazaud B. (2018). Muscle satellite cell cross-talk with a vascular niche maintains quiescence via VEGF and notch signaling. Cell. Stem Cell. 23, 530–543.e9. 10.1016/j.stem.2018.09.007 30290177 PMC6178221

[B221] Verma M. Asakura Y. Wang X. Zhou K. Ünverdi M. Kann A. P. (2024). Endothelial cell signature in muscle stem cells validated by VEGFA-FLT1-AKT1 axis promoting survival of muscle stem cell. eLife 13, e73592. 10.7554/eLife.73592 38842166 PMC11216748

[B222] Virtanen L. D'Ercole C. Giordani L. (2025). Across the space: applications of spatial transcriptomic technology in healthy and diseased muscle. Front. Cell. Dev. Biol. 13, 1656918. 10.3389/fcell.2025.1656918 41195339 PMC12583089

[B223] Waddell D. S. Baehr L. M. van den Brandt J. Johnsen S. A. Reichardt H. M. Furlow J. D. (2008). The glucocorticoid receptor and FoxO1 synergistically activate the skeletal muscle atrophy-associated MuRF1 gene. Am. J. Physiol. Endocrinol. Metab. 295, E785–E797. 10.1152/ajpendo.00646.2007 18612045 PMC2652500

[B224] Walsh C. J. Batt J. Herridge M. S. Mathur S. Bader G. D. Hu P. (2016). Transcriptomic analysis reveals abnormal muscle repair and remodeling in survivors of critical illness with sustained weakness. Sci. Rep. 6, 29334. 10.1038/srep29334 27411715 PMC4944143

[B225] Wang Y. X. Rudnicki M. A. (2012). Satellite cells, the engines of muscle repair. Nat. Rev. Mol. Cell. Biol. 13, 127–133. 10.1038/nrm3265 22186952

[B226] Wang H. Listrat A. Meunier B. Gueugneau M. Coudy-Gandilhon C. Combaret L. (2014). Apoptosis in capillary endothelial cells in ageing skeletal muscle. Aging Cell. 13, 254–262. 10.1111/acel.12169 24245531 PMC4331780

[B227] Wang C. Rabadan Ros R. Martinez-Redondo P. Ma Z. Shi L. Xue Y. (2021). *In vivo* partial reprogramming of myofibers promotes muscle regeneration by remodeling the stem cell niche. Nat. Commun. 12, 3094. 10.1038/s41467-021-23353-z 34035273 PMC8149870

[B228] Wieske L. Dettling-Ihnenfeldt D. S. Verhamme C. Nollet F. van Schaik I. N. Schultz M. J. (2015). Impact of ICU-acquired weakness on post-ICU physical functioning: a follow-up study. Crit. Care 19, 196. 10.1186/s13054-015-0937-2 25928709 PMC4427976

[B229] Wiig H. Swartz M. A. (2012). Interstitial fluid and lymph formation and transport: physiological regulation and roles in inflammation and cancer. Physiol. Rev. 92, 1005–1060. 10.1152/physrev.00037.2011 22811424

[B230] Wollersheim T. Woehlecke J. Krebs M. Hamati J. Lodka D. Luther-Schroeder A. (2014). Dynamics of myosin degradation in intensive care unit-acquired weakness during severe critical illness. Intensive Care Med. 40, 528–538. 10.1007/s00134-014-3224-9 24531339

[B231] Wong A. Garcia S. M. Tamaki S. Striedinger K. Barruet E. Hansen S. L. (2021). Satellite cell activation and retention of muscle regenerative potential after long-term denervation. Stem Cells 39, 331–344. 10.1002/stem.3316 33326654

[B232] Wosczyna M. N. Rando T. A. (2018). A muscle stem cell support group: coordinated cellular responses in muscle regeneration. Dev. Cell. 46, 135–143. 10.1016/j.devcel.2018.06.018 30016618 PMC6075730

[B233] Yang L. Xie L. Li M. Miao Y. Yang J. Chen S. (2025). Potential relationship between cuproptosis and sepsis-acquired weakness: an intermediate role for mitochondria. Front. Physiol. 16, 1520669. 10.3389/fphys.2025.1520669 40182687 PMC11965645

[B234] Yartseva V. Goldstein L. D. Rodman J. Kates L. Chen M. Z. Chen Y. J. (2020). Heterogeneity of satellite cells implicates delta1/NOTCH2 signaling in self-renewal. Cell. Rep. 30, 1491–1503.e6. 10.1016/j.celrep.2019.12.100 32023464

[B235] Yin H. Price F. Rudnicki M. A. (2013). Satellite cells and the muscle stem cell niche. Physiol. Rev. 93, 23–67. 10.1152/physrev.00043.2011 23303905 PMC4073943

[B236] Yoshihara I. Kondo Y. Okamoto K. Tanaka H. (2023). Sepsis-associated muscle wasting: a comprehensive review from bench to bedside. Int. J. Mol. Sci. 24, 5040. 10.3390/ijms24055040 36902469 PMC10003568

[B237] Yu Q. Song J. Yang L. Miao Y. Xie L. Ma X. (2024). A scoping review of preclinical intensive care unit-acquired weakness models. Front. Physiol. 15, 1423567. 10.3389/fphys.2024.1423567 PMC11480018 39416383

[B238] Yue F. Gu L. Qiu J. Oprescu S. N. Beckett L. M. Ellis J. M. (2025). Mitochondrial fatty acid oxidation regulates adult muscle stem cell function through modulating metabolic flux and protein acetylation. EMBO J. 44, 2566–2595. 10.1038/s44318-025-00397-1 40065099 PMC12048568

[B239] Zeng H. He X. Tuo Q. H. Liao D. F. Zhang G. Q. Chen J. X. (2016). LPS causes pericyte loss and microvascular dysfunction via disruption of SIRT3/angiopoietins/Tie-2 and HIF-2α/NOTCH3 pathways. Sci. Rep. 6, 20931. 10.1038/srep20931 26868537 PMC4751495

[B240] Zhang Z. S. Liu Y. Y. He S. S. Bao D. Q. Wang H. C. Zhang J. (2023). Pericytes protect rats and mice from sepsis-induced injuries by maintaining vascular reactivity and barrier function: implication of miRNAs and microvesicles. Mil. Med. Res. 10, 13. 10.1186/s40779-023-00442-2 36907884 PMC10010010

[B241] Zhou C. Wu L. Ni F. Ji W. Wu J. Zhang H. (2014). Critical illness polyneuropathy and myopathy: a systematic review. Neural Regen. Res. 9, 101–110. 10.4103/1673-5374.125337 25206749 PMC4146320

